# Integrated Machine Learning and Molecular Simulation-Guided Discovery of Novel Small-Molecule PD-L1 Inhibitors

**DOI:** 10.3390/ph19091439

**Published:** 2026-09-11

**Authors:** Mengjie Rui, Wenyan Liang, Kexin Chu, Jiukun Yuan, Ruojing Yang, Hangyu Dong, Chunlai Feng

**Affiliations:** 1 School of Pharmacy, Jiangsu University, 301 Xuefu Road, Zhenjiang 212013, China; mjrui@ujs.edu.cn (M.R.); 2212315013@stmail.ujs.edu.cn (W.L.); 2221815015@stmail.ujs.edu.cn (K.C.); 2212415045@stmail.ujs.edu.cn (J.Y.); 2222415036@stmail.ujs.edu.cn (R.Y.); 2222415042@stmail.ujs.edu.cn (H.D.); 2 Engineering and Technology Institute of Jiangsu University, Zhenjiang 212013, China; 3 Department of Obstetrics, Affiliated Hospital of Jiangsu University, No.438 Jiefang Road, Zhenjiang 212001, China; 4 Research Center for Intelligent Healthcare and Digital Health Technology, Affiliated Yixing People’s Hospital of Jiangsu University, Wuxi 214205, China

**Keywords:** PD-1/PD-L1 interaction, small-molecule inhibitors, machine learning, genetic algorithm, molecular dynamics simulation, antitumor immunity

## Abstract

**Background/Objectives**: The programmed death-1/programmed death-ligand 1 (PD-1/PD-L1) immune checkpoint is a key therapeutic target in cancer immunotherapy, but small-molecule inhibition remains challenging due to its shallow and dynamic interaction interface. This study aimed to develop an artificial intelligence (AI)-guided workflow to identify novel small-molecule inhibitors targeting the PD-L1 dimer interface. **Methods**: A combined computational and experimental approach was established. A support vector regression-genetic algorithm (SVR-GA) model was trained on a dataset of 1385 known PD-L1 inhibitors to predict activity and guide molecular generation. From 470 AI-generated candidates, docking and molecular dynamics (MD) simulations were used for virtual screening. Selected compounds were synthesized and evaluated for PD-1/PD-L1 binding disruption using homogeneous time-resolved fluorescence (HTRF) assays. Cytotoxicity was tested in MDA-MB-231 and 4T1 cell monocultures, and in vivo efficacy was assessed in an immunocompetent 4T1 tumor model. **Results**: Two hits, PD-L1-Ser and PD-L1-Ser-OEt, were identified. Both disrupted PD-1/PD-L1 binding in HTRF assays, with PD-L1-Ser-OEt showing higher potency (IC_50_ = 0.2068 μM). Both compounds exhibited limited direct cytotoxicity in cancer cell monocultures, suggesting an immune-mediated mechanism. In the 4T1 syngeneic mouse model, both inhibitors suppressed tumor growth without causing body weight loss. PD-L1-Ser-OEt demonstrated superior antitumor efficacy and elevated serum levels of IFN-γ and IL-4. **Conclusions**: This AI-guided workflow combining machine-learning-based molecular generation with structure validation is feasible for discovering PD-L1 dimer-interface inhibitors. PD-L1-Ser-OEt represents a promising lead compound for further development as an immune checkpoint inhibitor.

## 1. Introduction

Programmed death-1 (PD-1) is an inhibitory receptor expressed on activated T cells, whereas programmed death-ligand 1 (PD-L1) is frequently upregulated on tumor cells and antigen-presenting cells [1,2]. Engagement of PD-1 by PD-L1 suppresses cytotoxic T-cell function, decreases the production of effector cytokines such as interferon-gamma (IFN-γ), tumor necrosis factor-alpha (TNF-α), and interleukin-2 (IL-2), and promotes T-cell exhaustion, thereby facilitating tumor immune evasion and disease progression. Consequently, blockade of the PD-1/PD-L1 axis has become a central strategy in cancer immunotherapy [3,4,5].

Monoclonal antibodies targeting PD-1/PD-L1 have achieved major clinical success, but antibody-based therapies can be limited by immune-related adverse events, complex administration, high manufacturing costs, and suboptimal penetration into some solid tumors [6,7]. Small-molecule inhibitors could complement antibody therapeutics because their physicochemical properties can be tuned, they may permit oral or other flexible routes of administration, and they may access anatomical sites that are difficult for large biologics to reach [8,9]. However, discovering potent and drug-like small-molecule PD-L1 inhibitors remains challenging because the PD-1/PD-L1 interface is relatively shallow, dynamic, and poorly suited to conventional ligand design [10,11].

Currently, several small-molecule PD-L1 inhibitors have been reported, with BMS-series compounds (e.g., BMS202, BMS1001, BMS1166) representing the most well-characterized class. These ligands bind at the PD-L1 dimer interface and induce dimer stabilization [12,13]. Among them, BMS202 is a prototypical inhibitor that stabilizes the PD-L1 dimer and blocks PD-1 binding with low-micromolar to nanomolar biochemical potency [14]. Under the reported HTRF assay conditions, their inhibitory activities are approximately 18 nM, 2.25 nM, and 1.4 nM, respectively; their structures and reported values are summarized in Supplementary Figure S1. The clinically evaluated oral PD-L1 inhibitor INCB086550 further supports the translational potential of this mechanism. Despite these advances, the structural diversity of PD-L1 small-molecule inhibitors remains limited, and many reported compounds suffer from poor physicochemical properties or lack of in vivo efficacy [9,15]. For instance, BMS202 has poor aqueous solubility and low oral bioavailability, which limit its systemic application. More importantly, the discovery of most existing inhibitors relies on high-throughput screening or modification of known scaffolds, lacking efficient and generalizable de novo design strategies, which slows the identification of new chemotypes. Therefore, there is an urgent need to develop new chemotypes and more efficient discovery strategies to expand the repertoire of PD-L1-targeting small molecules and to improve the translation efficiency from computational prediction to experimental validation.

Recent advances in artificial intelligence (AI)-assisted drug discovery have created new opportunities for data-driven activity prediction and efficient exploration of chemical space. In this study, we established an integrated discovery pipeline that combines support vector regression (SVR), genetic algorithm (GA)-enabled de novo molecular generation, structure-based docking, and molecular dynamics (MD) simulations to identify novel PD-L1 dimer-interface inhibitors [16,17,18]. An SVR model trained on a curated dataset of PD-1/PD-L1 small-molecule inhibitors was used as a fitness function to guide GA-driven molecular evolution and candidate prioritization [19]. Docking and MD simulations based on the PD-L1 dimer crystal structure were subsequently applied as orthogonal filters to evaluate binding modes, key interfacial interactions, and complex stability [20,21].

The selected candidates were synthesized and experimentally evaluated using a homogeneous time-resolved fluorescence (HTRF) assay to quantify PD-1/PD-L1 blockade, followed by in vitro cytotoxicity testing and in vivo efficacy assessment in a 4T1 tumor-bearing mouse model. By integrating ligand-based prediction, physics-based structural filtering, and experimental validation into a single workflow, this study provides a new chemotype for PD-L1 inhibition and validates an integrated computational-experimental pipeline, thereby offering a practical route and mechanistically informed starting points for next-generation immune checkpoint therapeutics.

## 2. Results

### 2.1. SVR-GA Integrated Model for Activity Prediction and De Novo Design of PD-L1 Inhibitors

**Figure 1:**
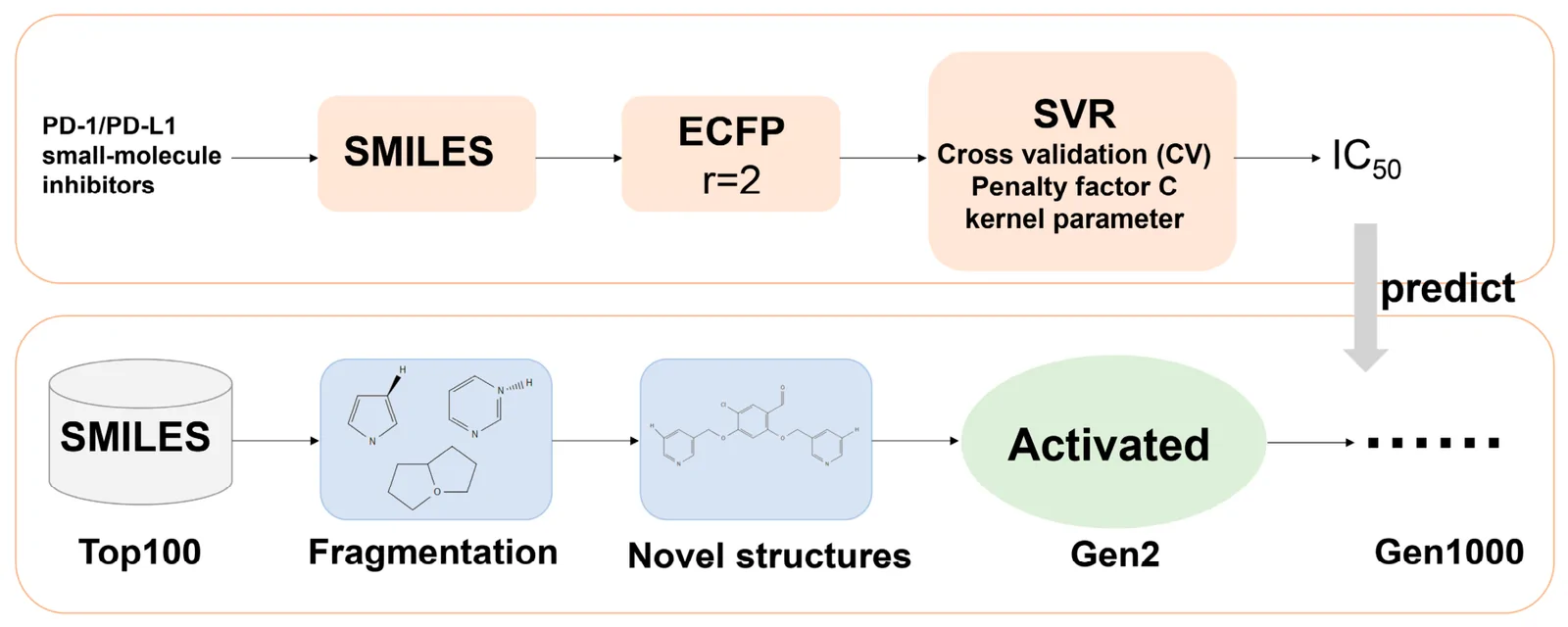
Overview of the SVR-GA integrated platform for PD-L1 inhibitor discovery. ECFP fingerprints (radius = 2) were generated from SMILES representations of PD-1/PD-L1 small-molecule inhibitors and used to construct an SVR model for IC_50_ prediction, incorporating cross-validation, the penalty factor C, and kernel parameters. The optimized model was then used as a fitness function in a genetic algorithm (GA)-based molecular design workflow. Starting from the top 100 most active compounds, molecular fragments were recombined to generate new structures, which were then evaluated by the SVR model for activity prediction and candidate prioritization.

An integrated SVR-GA model for activity prediction and de novo molecular design of PD-L1 inhibitors was constructed (Figure 1). A structure-activity dataset comprising 1385 PD-1/PD-L1 small-molecule inhibitors with curated chemical structures and IC_50_ values was assembled. Compounds were randomly divided into a training set (*n* = 1108) and an independent test set (*n* = 277). ECFP fingerprints were generated as molecular descriptors, and IC_50_ values were log-transformed to pIC_50_.

During model development, a polynomial-kernel configuration (C = 0.1, γ = 0.1) evaluated under three-fold cross-validation yielded an RMSE of 0.484 and an R^2^ of 0.755, whereas an RBF configuration (C = 10, γ = 0.01) evaluated under seven-fold cross-validation yielded an RMSE of 0.634 and an R^2^ of 0.806. Because these metric pairs were obtained under different resampling settings, they were not interpreted as a direct comparison of predictive performance on a common evaluation set. The RBF configuration was retained as the fitness function for downstream GA-based candidate ranking.

The GA workflow comprised population initialization (top 100 active compounds), fragment extraction, crossover-based offspring generation, and iterative evolution. After 1000 generations, 470 structurally diverse candidate molecules with predicted IC_50_ below 8 nM were generated and subsequently subjected to structure-based screening. Because no independent external or scaffold-stratified validation set and no formal applicability-domain filter were used, these numerical predictions were treated as internal ranking scores rather than experimentally calibrated potency estimates.

### 2.2. Molecular Dynamics-Guided Identification of Key PD-L1 Binding Hot Spots

**Figure 2:**
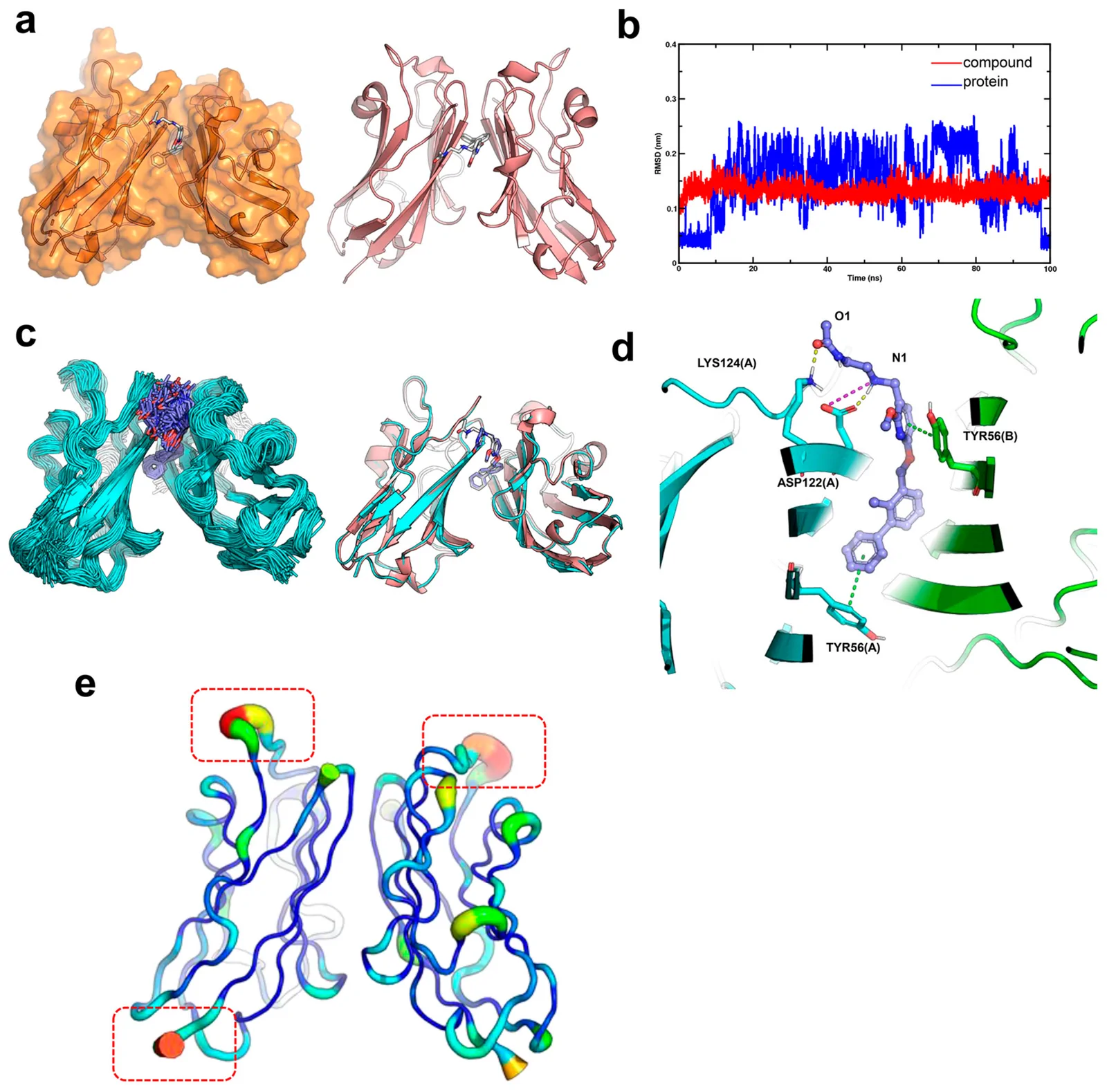
MD analysis of the PD-L1 dimer bound to BMS202. (**a**) Crystal structure of the human PD-L1 dimer in complex with BMS202 (PDB ID: 5J89). In the surface representation (**left**), the two PD-L1 protomers are shown as dark- and light-orange cartoons beneath a semitransparent orange molecular surface, and BMS-202 is shown as sticks colored by atom type. In the cartoon representation (**right**), the two protomers are shown in salmon and light gray, and BMS-202 is shown as atom-colored sticks. Arrow-shaped ribbons denote beta-strands, helical ribbons denote alpha-helices, and thin tubes denote loops/coils. (**b**) RMSD profiles of the protein (blue) and BMS-202 (red) over 100 ns. RMSD trajectory of the PD-L1-BMS202 complex. (**c**) Conformational sampling and structural superposition. Left, sampled protein conformations are shown as cyan ribbons and sampled BMS-202 conformations as purple/blue/red sticks. Right, the initial crystallographic structure is shown in salmon and the final MD snapshot in cyan, with the corresponding ligand conformations shown as sticks. (**d**) Representative interactions at the dimer interface. Chain A is shown in cyan, chain B in green, and BMS-202 in lavender. Yellow dashed lines denote hydrogen bonds, the magenta dashed line denotes an ionic/salt-bridge contact, and green dashed lines denote aromatic pi interactions. (**e**) RMSF values mapped onto the PD-L1 dimer using a blue-to-red color scale and increasing tube thickness, where blue/thin regions are relatively rigid and yellow-to-red/thick regions are more flexible. Red dashed boxes highlight the most flexible loop regions, including the Lys46-Trp57 and Met115-Lys124 segments.

To characterize binding-site dynamics and define key interaction residues for structure-guided screening, MD simulations were first performed on the PD-L1 dimer-BMS202 co-crystal structure (PDB ID: 5J89; Figure 2a).

#### 2.2.1. Structural Stability Assessed by RMSD

Although the 100 ns trajectory was not enough to interpret the complete equilibration, RMSD analysis demonstrated BMS202 remained within the crystallographic interfacial pocket after approximately 15 ns (Figure 2b). Thereafter, only modest fluctuations were observed, supporting a stable ligand-protein complex. Inspection of the trajectory showed that BMS202 remained in the original pocket throughout the simulation (Figure 2c), and the final MD conformation aligned closely with the crystallographic pose, supporting the reliability of subsequent interaction and energetic analyses.

#### 2.2.2. Hydrogen-Bond and Noncovalent Interaction Profiling

Hydrogen-bond analysis revealed persistent interactions between BMS202 and the PD-L1 dimer (Table 1). In particular, the imino hydrogen of BMS202 acted as a donor to the ASP122 side-chain oxygen with an occupancy of 63.14%. In addition, LYS124 formed a hydrogen bond with BMS202 with an occupancy of 62.49%, indicating that ASP122 and LYS124 contribute to stabilization of the complex.

The final MD conformation also revealed a salt bridge involving ASP122 and π-π stacking interactions with TYR56 residues from both PD-L1 chains (Figure 2d). Together, these interactions identify TYR56, ASP122, and LYS124 as key residues that stabilize binding at the PD-L1 dimer interface.

#### 2.2.3. MM/PBSA Energetic Decomposition

MM/PBSA analysis of the BMS202-PD-L1 trajectory yielded a mean calculated interaction-energy estimate of −167.310 ± 11.913 kJ/mol (Table 2). Within this computational framework, van der Waals (−261.960 ± 11.120 kJ/mol) and electrostatic (−42.842 ± 8.676 kJ/mol) terms contributed favorably, whereas polar solvation opposed complex formation. Notably, these values represent model-dependent energetic summaries of the sampled trajectory are not experimentally measured binding free energies or direct measures of thermodynamic affinity.

Per-residue decomposition identified several residues with favorable calculated contributions, including Thr20, Ile54, Tyr56, Met115, Ser117, Ala121, Asp122, Tyr123, and Lys124 in chain A, as well as Val55, Trp57, Ile116, and Tyr118 in chain B (Supplementary Figure S2A,B). Together with the hydrogen-bond and structural analyses, these calculations suggest that the regions spanning ILE54-TRP57 and MET115-LYS124 form the core pocket for small-molecule recognition and provide a rational basis for selecting flexible docking residues.

#### 2.2.4. Protein Flexibility Assessed by RMSF

RMSF analysis was performed to identify flexible regions in the PD-L1 dimer. As shown in Supplementary Figure S2C,D, residues highlighted in green exhibited low RMSF values and thus relatively rigid behavior, whereas residues highlighted in red displayed larger fluctuations, indicating greater flexibility.

Mapping RMSF values onto the protein structure (Figure 2e) identified pronounced flexibility in the loop regions spanning LYS46-TRP57 and MET115-LYS124. Together with the hydrogen-bond and MM/PBSA analyses, these data support TYR56 and ASP122 as key residues for subsequent structure-based screening and docking.

### 2.3. Flexible Docking and Hit Selection

**Figure 3:**
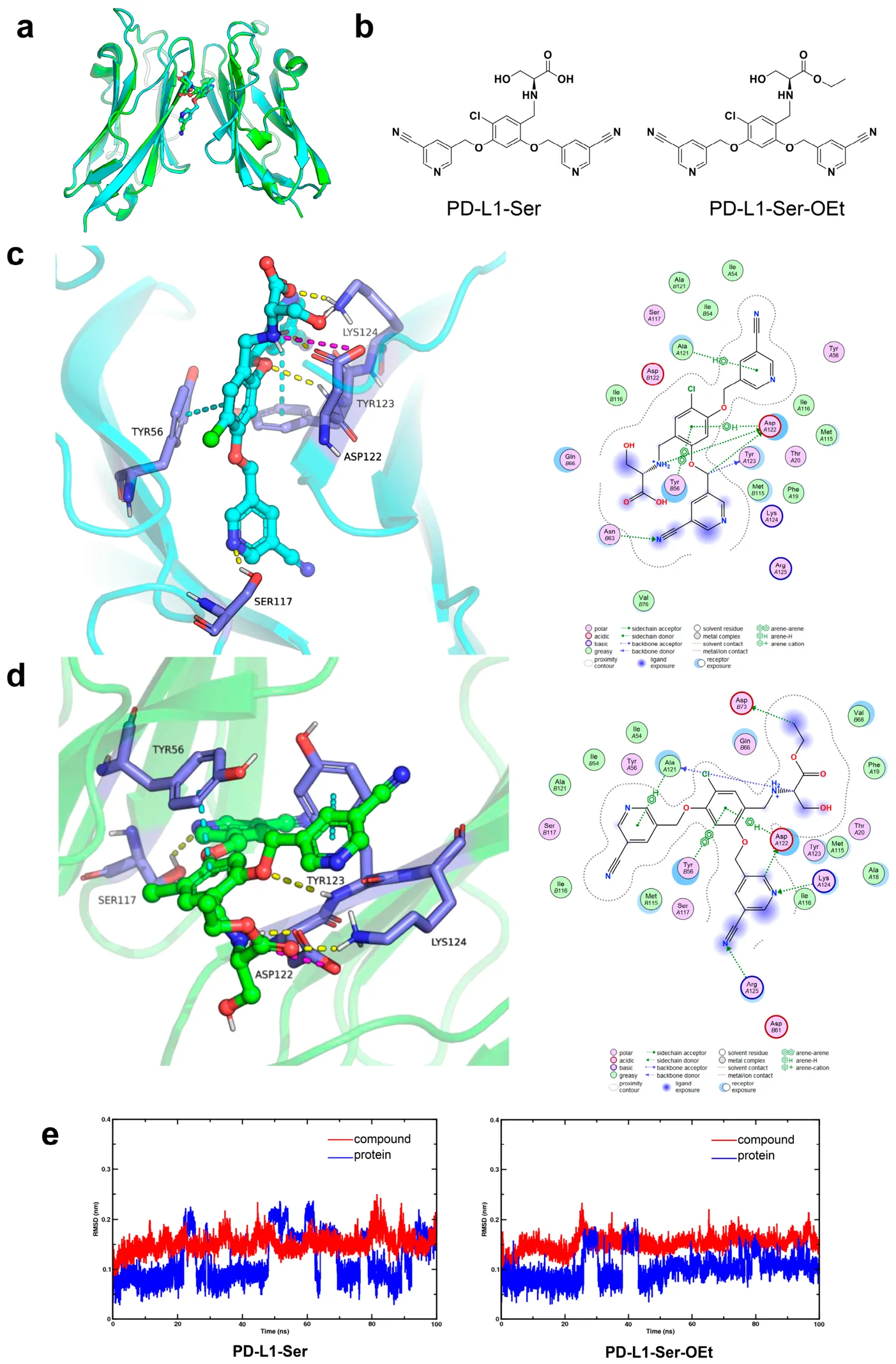
Docking and MD validation of PD-L1-Ser and PD-L1-Ser-OEt. (**a**) Superposition of the crystallographic human PD-L1-BMS-202 complex and the redocked complex. The two proteins are shown in green and cyan, and the corresponding BMS-202 poses are shown as sticks. (**b**) Chemical structures of the selected candidate compounds PD-L1-Ser (**left**) and PD-L1-Ser-OEt (**right**). (**c**,**d**) Predicted three-dimensional binding modes (**left**) and two-dimensional interaction maps (**right**) of PD-L1-Ser and PD-L1-Ser-OEt, respectively. In the three-dimensional views, the PD-L1 dimer is shown as a pale cyan or pale green cartoon, the candidate ligand as cyan or green sticks/balls, and selected pocket residues as lavender sticks. Ligand/residue atoms are colored conventionally (oxygen, red; nitrogen, blue; chlorine, green). Yellow dashed lines denote hydrogen bonds, magenta dashed lines denote ionic/salt-bridge contacts, and green dashed lines denote aromatic interactions. In the two-dimensional maps, residue circles are colored by physicochemical class (polar, purple; acidic, red; basic, blue; hydrophobic/greasy, green), and interaction symbols follow the embedded legend, including side-chain/backbone donor or acceptor contacts, proximity contours, solvent/receptor exposure, and aromatic interactions. (**e**) RMSD trajectories of the PD-L1 dimer in complex with PD-L1-Ser and PD-L1-Ser-OEt over 100 ns.

Flexible docking was performed to prioritize GA-generated candidates for experimental validation. Redocking BMS202 into the human PD-L1 dimer produced a heavy-atom RMSD of 0.783 Å relative to the crystallographic pose (Figure 3a), demonstrating pose-recovery capability for this reference complex, validating the docking protocol. TYR56 and ASP122 were treated as flexible residues on the basis of the MD analysis.

Using this protocol, all 470 candidates were docked. A score threshold of −12 kcal/mol retained 26 compounds (5.5% of the generated set) and was used as a pragmatic enrichment cutoff to make subsequent visual inspection and synthetic assessment tractable. After consideration of docking score, key residue interactions, hydrogen-bond patterns, and synthetic feasibility, two representative candidates were selected and designated PD-L1-Ser and PD-L1-Ser-OEt (Figure 3b).

Docking reproducibility was further evaluated in three independent runs using random seeds of 101, 202, and 303. Three established PD-L1 inhibitors were included as reference compounds. The resulting mean best-ranked scores were −12.847 ± 0.067 kcal/mol for BMS-202, −12.667 ± 0.029 kcal/mol for BMS-1001, and −13.313 ± 0.047 kcal/mol for BMS-1166. PD-L1-Ser and PD-L1-Ser-OEt yielded corresponding values of −12.352 ± 0.181 and −12.479 ± 0.027 kcal/mol, respectively (Supplementary Table S1). The narrow variation among independent runs indicated good reproducibility of the Vina search under the predefined docking conditions. The candidate compounds also occupied the same general nominal score range as the BMS-series reference ligands.

PD-L1-Ser was predicted to form π-π stacking interactions between its phenyl ring and TYR56, a hydrogen bond between the nitrogen atom of the cyanopyridine moiety and SER117, and additional aromatic interactions with TYR123 (Figure 3c). The secondary amine also formed a hydrogen bond and salt bridge with ASP122, whereas the carboxylate engaged LYS124 through a salt bridge, together supporting a stable complex with PD-L1.

PD-L1-Ser-OEt exhibited a broadly similar binding mode within the PD-L1 dimer interface (Figure 3d). However, because the esterified side chain may alter desolvation and packing effects, MD simulation was used to further assess the stability of this complex.

Overall, the docking analysis provided a reproducible structure-based prioritization step and generated plausible binding hypotheses for subsequent MD simulation and experimental evaluation.

### 2.4. MD Assessment of the Candidate PD-L1 Complexes

To examine the behavior of the predicted PD-L1-Ser and PD-L1-Ser-OEt complexes under explicit-solvent conditions, 100 ns MD simulations were performed from their respective docked conformations. Both compounds remained within the proposed PD-L1 interfacial region during the simulated time window, although appreciable fluctuations were observed in both protein and ligand trajectories (Figure 3e). The simulations were therefore used to assess persistence of the modeled binding configurations rather than to establish complete conformational equilibration.

MM/PBSA analysis yielded mean calculated interaction-energy estimates of −114.287 ± 13.214 kJ/mol for PD-L1-Ser and −119.157 ± 14.887 kJ/mol for PD-L1-Ser-OEt (Table 3). Both calculations contained favorable van der Waals and electrostatic components that were partially offset by polar-solvation penalties.

Importantly, the approximately 4.9 kJ/mol difference between the mean MM/PBSA estimates was small relative to the corresponding trajectory-associated variability. These calculations therefore do not provide a quantitative basis for ranking the binding affinities of PD-L1-Ser and PD-L1-Ser-OEt and cannot explain the difference in their experimentally measured HTRF potency. Instead, the simulations support the structural plausibility and persistence of the proposed binding modes over the sampled trajectories.

### 2.5. Cross-Species Context for the 4T1 Model

The HTRF assay and the primary docking and MD analyses in this study were performed using human proteins. To evaluate the cross-species relevance of these findings to the murine 4T1 model, we compared the PD-1-binding IgV domains of human PD-L1 and mouse PD-L1 using the experimentally determined structures 5J89 and 6SRU.

Published analysis reported 69.4% sequence identity and 87.6% similarity between the human and mouse PD-L1 IgV domains [22]. Consistent with this high degree of conservation, structural superposition showed closely related global IgV folds. Nevertheless, several local differences were observed within or adjacent to the proposed small-molecule-binding region. Human Ile54, Asn63, Arg113, and Met115 corresponded to mouse Val54, Gln63, Cys113, and Ile115, respectively. Tyr56 was conserved at the sequence level, but its side chain adopted a different orientation in the mouse structure, with an approximately 7 Å displacement of the terminal hydroxyl group reported between the two structures [22]. The longer Gln63 side chain in mouse PD-L1 was positioned near Tyr56, whereas the Met115-to-Ile substitution introduced a β-branched side chain into the hydrophobic region. These differences indicate that similar global folding does not ensure conservation of local pocket shape or conformational flexibility (Supplementary Figure S3).

We next performed docking using a mouse PD-L1 dimer model generated by superposing two copies of the monomeric mouse PD-L1 (PDB ID: 6SRU) structure onto the two ligand-associated human PD-L1 protomers in 5J89. The top-ranked docking scores were −6.41 kcal/mol for BMS202, −6.40 kcal/mol for PD-L1-Ser, and −5.90 kcal/mol for PD-L1-Ser-OEt. In each case, the top-ranked pose was displaced from the crystallographic human BMS202 position and did not fully reproduce the canonical orientation observed in 5J89 (Supplementary Figure S4).

### 2.6. Chemical Synthesis and Structural Characterization

**Figure 4:**
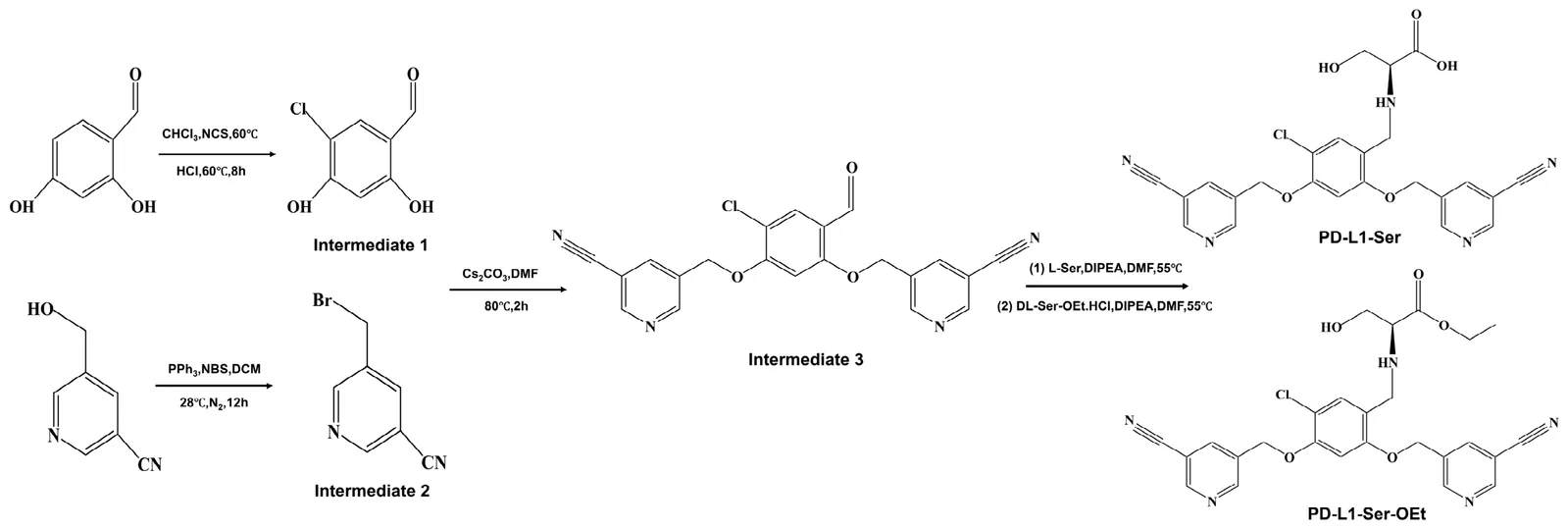
Synthetic routes to the hit compounds PD-L1-Ser and PD-L1-Ser-OEt.

The target PD-L1 inhibitor candidates, PD-L1-Ser and PD-L1-Ser-OEt, were synthesized following the route outlined in Figure 4. Starting from commercially available 2,4-dihydroxybenzaldehyde, electrophilic chlorination with N-chlorosuccinimide and concentrated HCl in chloroform at 60 °C afforded 5-chloro-2,4-dihydroxybenzaldehyde (intermediate **1**). Separately, 5-(hydroxymethyl)nicotinonitrile was treated with triphenylphosphine and N-bromosuccinimide in dichloromethane at 28 °C to give 5-(bromomethyl) nicotinonitrile (intermediate **2**). Williamson etherification of intermediates **1** and **2** using Cs_2_CO_3_ in DMF at 80 °C produced the dialdehyde intermediate **3** (MS spectrum shown in Supplementary Figure S5a). Finally, reductive amination of intermediate **3** with L-serine or DL-serine ethyl ester hydrochloride in the presence of NaBH_3_CN and DIPEA in DMF/H_2_O at 55 °C furnished PD-L1-Ser and PD-L1-Ser-OEt, respectively, in overall yields of 54.3% and 67.2%.

**Figure 5:**
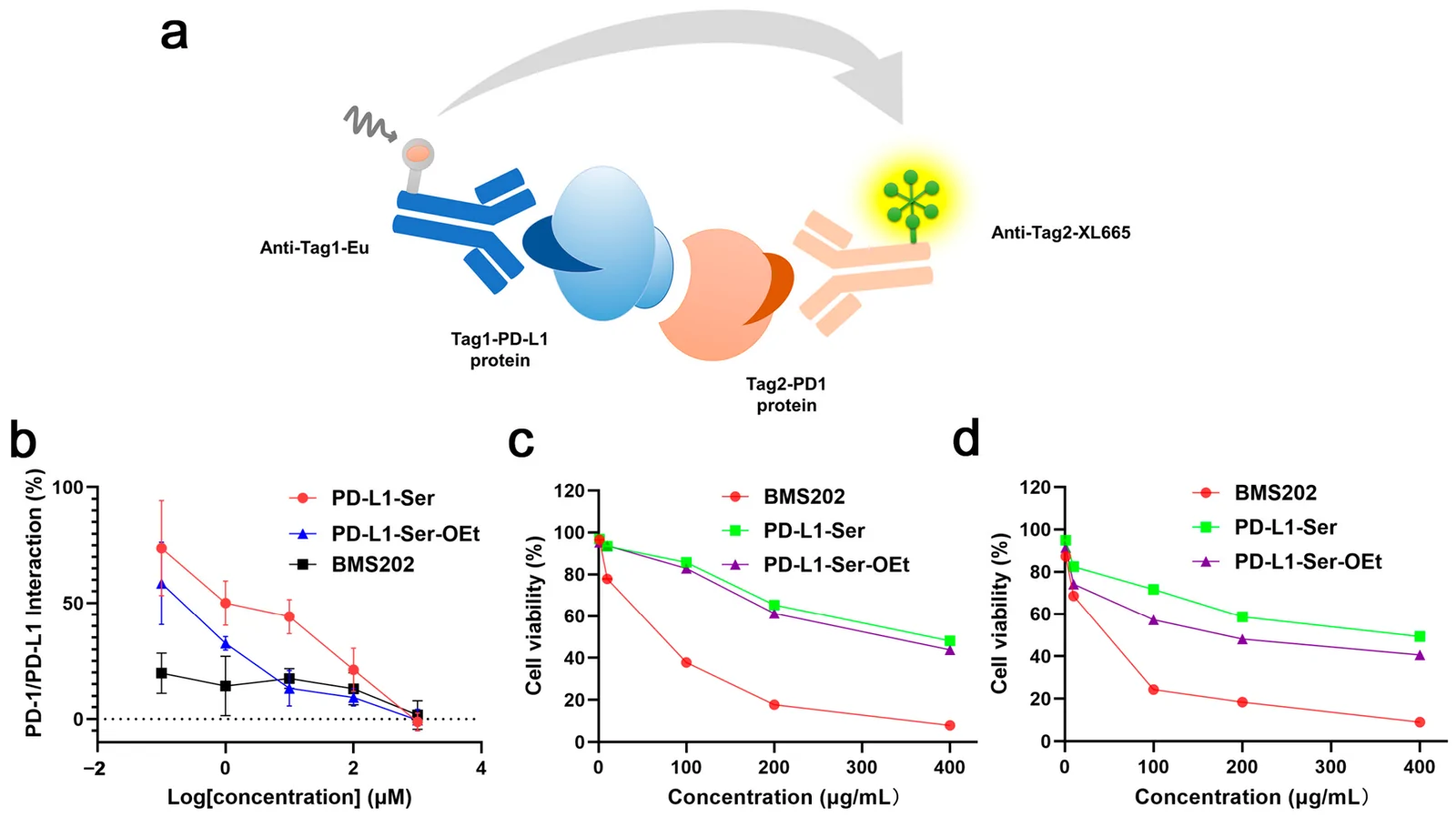
In vitro activity evaluation. (**a**) Schematic of the HTRF-based PD-1/PD-L1 inhibition assay. PD-L1-Tag1 and PD-1-Tag2 are labeled with anti-Tag1-Eu (donor) and anti-Tag2-XL665 (acceptor). Upon PD-1/PD-L1 binding, Eu and XL665 come close; laser excitation of Eu leads to FRET and XL665 emission. Compound that blocks the interaction prevents FRET, resulting in no XL665 signal. This setup measures compound inhibition. (**b**) Inhibitory effects of PD-L1-Ser and PD-L1-Ser-OEt on PD-1/PD-L1 binding. (**c**) Viability of MDA-MB-231 cells after treatment with the candidate compounds. (**d**) Viability of 4T1 cells after treatment with the candidate compounds.

The structures of all newly synthesized intermediates and final compounds were confirmed by high-resolution mass spectrometry (HRMS), ^1^H NMR and ^13^C NMR spectroscopy (Supplementary Figure S5). A schematic representation of the synthetic route and the corresponding characterization data are presented in Figure 4 and Figure 5.

### 2.7. In Vitro PD-1/PD-L1 Blockade Assessed by HTRF

HTRF assays were used to quantify the PD-1/PD-L1 binding level in the presence of PD-L1-Ser and PD-L1-Ser-OEt (Figure 5a). BMS202 served as a positive control, showing a binding level of 14.29% of the control at 1 μM. Both candidate compounds decreased the binding level in a concentration-dependent manner across the tested range (Table 4). At 1 μM, PD-L1-Ser-OEt exhibited a binding level of 32.59% of the control, which was markedly lower than the 49.98% observed for PD-L1-Ser (Figure 5b). Across the dose range, PD-L1-Ser-OEt consistently showed stronger binding disruption than PD-L1-Ser and exhibited a lower IC_50_ (0.2068 μM versus 1.852 μM), indicating improved potency in blocking the PD-1/PD-L1 interaction. At the highest tested concentrations, the binding was nearly completely abolished.

### 2.8. In Vitro Cytotoxicity Against Breast Cancer Cell Lines

The antiproliferative activity of PD-L1-Ser and PD-L1-Ser-OEt was evaluated in MDA-MB-231 and 4T1 cells using CCK-8 assays (Figure 5c,d).

In MDA-MB-231 cells, BMS202 showed the strongest activity with an IC_50_ of 47.68 µg/mL, whereas PD-L1-Ser-OEt was modestly more active than PD-L1-Ser, with IC_50_ values of 338.5 µg/mL and 402.2 µg/mL, respectively (Figure 5c, Table 5). In 4T1 cells, BMS202 again showed the greatest cytotoxicity (IC_50_ = 27.78 µg/mL), while PD-L1-Ser-OEt (IC_50_ = 174.4 µg/mL) remained more active than PD-L1-Ser (IC_50_ = 303.6 µg/mL) (Figure 5d, Table 6).

Overall, both candidates showed substantially weaker direct cytotoxicity than BMS202. This result indicated that strong direct tumor-cell killing was not a dominant feature of PD-L1-Ser and PD-L1-Ser-OEt at the tested concentrations. In addition, these assays lack functional immune effector cells, they do not evaluate checkpoint-mediated immune activity. Therefore, the subsequent in vivo study was applied to determine the activities of PD-L1-Ser and PD-L1-Ser-OEt and investigate the underlying mechanism.

### 2.9. In Vivo Antitumor Efficacy in a 4T1 Tumor-Bearing Mouse Model

**Figure 6:**
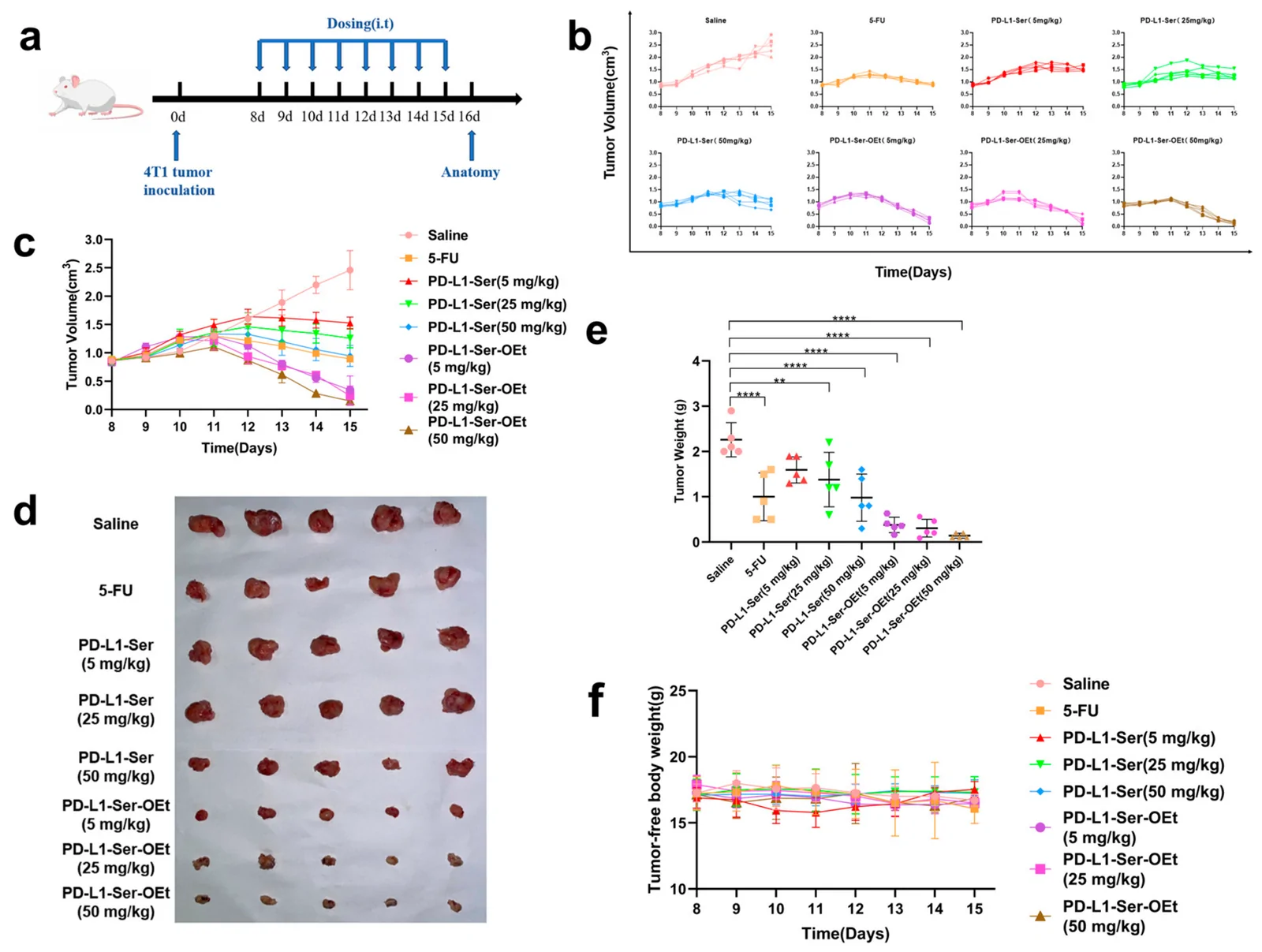
In vivo antitumor efficacy and tolerability in 4T1 tumor-bearing mice. (**a**) In vivo dosing regimen. (**b**,**c**) Changes in tumor volume during treatment. (**d**) Representative tumor morphology at necropsy. (**e**) Terminal tumor weights. (** *p* < 0.01, and **** *p* < 0.0001 versus Saline group). (**f**) Changes in body weight during treatment.

In the 4T1 tumor-bearing mouse model, tumor growth was suppressed in all treatment groups relative to saline controls over the 8-day observation period (Figure 6b,c). The overall efficacy trend was high-dose PD-L1-Ser-OEt > medium-dose PD-L1-Ser-OEt > low-dose PD-L1-Ser-OEt > 5-FU > high-dose PD-L1-Ser > medium-dose PD-L1-Ser > low-dose PD-L1-Ser. Across dose levels, PD-L1-Ser-OEt consistently produced stronger tumor suppression than PD-L1-Ser and outperformed the 5-FU control. The non-tumor-bearing healthy control group served solely as a physiological reference and was therefore not included in analyses of tumor volume, terminal tumor weight, or tumor-inhibition rate.

Terminal tumor weight and inhibition rate were consistent with the tumor-volume data (Table 7; Figure 6d,e). The high-dose PD-L1-Ser group showed a tumor inhibition rate comparable to that of 5-FU. Notably, the high-dose PD-L1-Ser-OEt group achieved an inhibition rate of 93.81%, whereas the medium- and low-dose groups achieved 86.46% and 83.27%, respectively, indicating a strong antitumor response in vivo.

No marked body-weight loss was observed relative to saline controls during the short treatment period (Figure 6f), suggesting acceptable short-term tolerability under the conditions tested. Overall, PD-L1-Ser-OEt showed superior in vivo efficacy and warrants further evaluation in longer-term efficacy, mechanistic, and safety studies.

### 2.10. Serum Cytokine Profiling by ELISA

**Figure 7:**
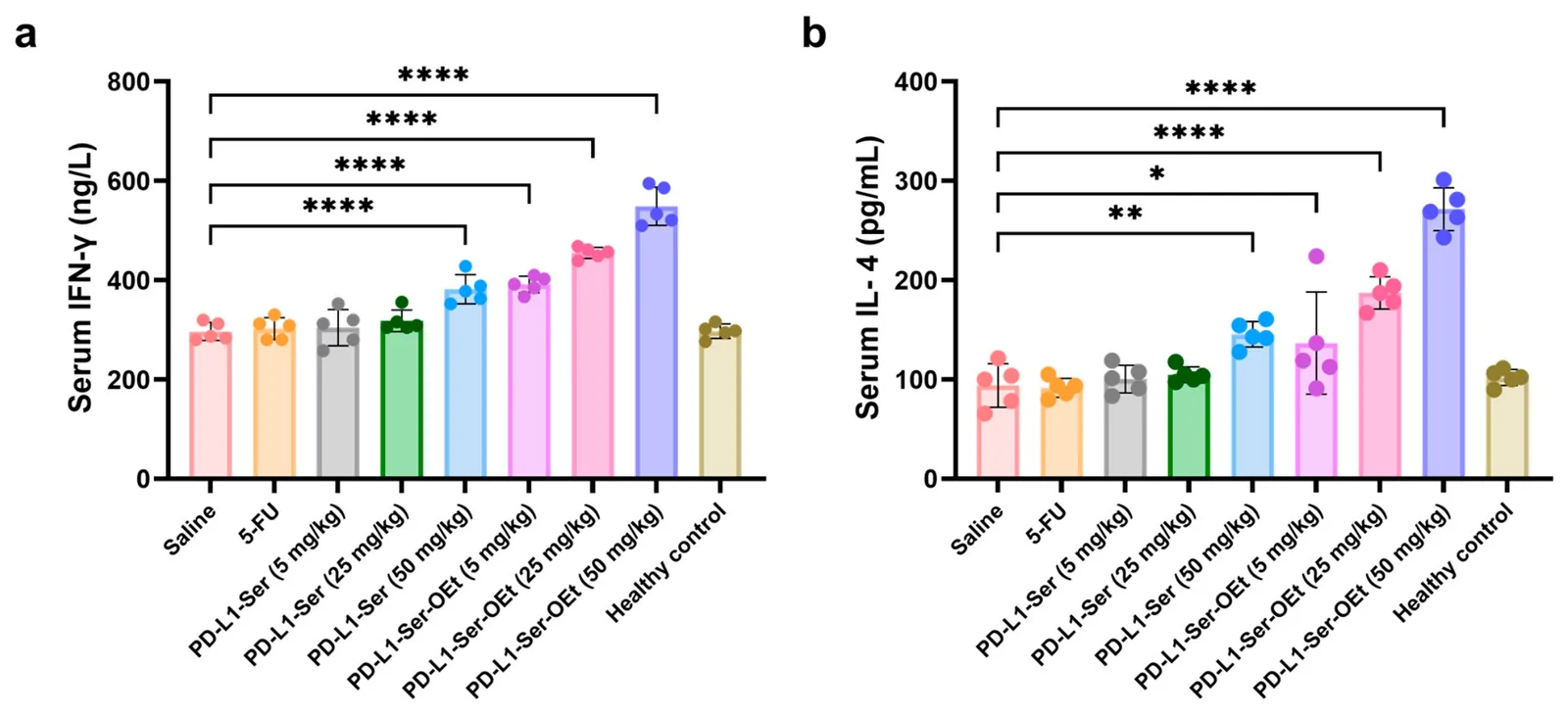
Serum cytokine levels in 4T1 tumor-bearing mice. (**a**) IFN-γ levels in serum samples (*n* = 5). (**b**) IL-4 levels in serum samples (*n* = 5) (* *p* < 0.05, ** *p* < 0.01, and **** *p* < 0.0001 versus saline group).

Serum IFN-γ and IL-4 concentrations were evaluated in the tumor-bearing treatment groups together with the non-tumor-bearing healthy control group (Figure 7a,b; Table 8). The vehicle-treated tumor-bearing group served as the primary pharmacodynamic comparator, whereas the healthy control group provided a physiological reference for circulating cytokine concentrations. Dose-related increases in both cytokines were observed in several candidate-treated groups, with the highest mean concentrations detected in the high-dose PD-L1-Ser-OEt group. The candidate compounds significantly promote cytokine release, which might be associated with the potential immune modulation with PD-1/PD-L1 blockade. However, cytokines were secondary systemic biomarkers, and their changes might be influenced by tumor burden and other treatment-associated systemic responses. Accordingly, the observed cytokine changes can partly demonstrate activation of tumor-infiltrating T cells and PD-1/PD-L1 signaling.

Together with the HTRF evidence of PD-1/PD-L1 blockade (Figure 5b; Table 4), the modest direct cytotoxicity observed in MDA-MB-231 and 4T1 monocultures (Figure 5c,d; Table 5 and Table 6), and the marked tumor suppression in immunocompetent 4T1 mice (Figure 6b–e; Table 7) with limited short-term body-weight change (Figure 6f), these cytokine data suggest that PD-L1-Ser and PD-L1-Ser-OEt exert antitumor effects at least partly through anti-tumor immune activation. Overall, the integrated AI-assisted pipeline enabled prioritization of two synthetically accessible PD-L1 dimer-interface inhibitors for experimental validation.

## 3. Discussion

In this study, we assembled a structure-activity dataset of 1385 PD-1/PD-L1 small-molecule inhibitors and established an integrated discovery platform that combines machine-learning activity prediction with structure-guided molecular simulation. Using this pipeline, we designed and prioritized two synthetically accessible PD-L1 candidates whose stable association with PD-L1 was supported by MD simulations and then validated experimentally. Taken together, these results support the feasibility of AI-guided, simulation-informed workflows for accelerating the discovery of small-molecule immune checkpoint inhibitors.

Small-molecule inhibition of PD-L1 is structurally distinct from conventional occupancy of a well-defined enzyme active site. BMS-series inhibitors bind within the PD-L1 dimer interface and stabilize a ligand-induced dimeric state that occludes the PD-1 binding surface [23,24]. Consistent with this mechanism, analysis of the BMS-202-PD-L1 complex highlighted an interfacial region centered on Tyr56 and the Met115-Lys124 segment, with contributions from hydrophobic packing, aromatic interactions, and polar contacts (Table 1; Figure 2d,e). Hydrogen-bond analysis, RMSF profiling, and MM/PBSA per-residue decomposition converged on this region as a structurally relevant site for candidate prioritization. Redocking of BMS-202 reproduced its crystallographic pose with an RMSD of 0.783 Å. In addition, three independent Vina runs using different random seeds produced only limited variation in docking scores for BMS-202, BMS-1001, BMS-1166, PD-L1-Ser, and PD-L1-Ser-OEt, supporting reproducibility of the stochastic docking search under the predefined conditions. These analyses establish pose and search reproducibility, whereas the Vina scores themselves were used as structure-based prioritization metrics rather than quantitative measures of binding affinity.

A methodological strength of the study is the sequential integration of ligand-based and structure-based filters rather than reliance on a single computational score. The SVR model provided an efficient fitness function for exploring chemical space during GA evolution, after which docking and explicit-solvent MD were used to examine compatibility with the PD-L1 dimer-interface pocket and persistence of the proposed binding configurations. The model was evaluated using an internal hold-out set generated from the same curated dataset; therefore, its current performance primarily supports candidate ranking within this chemical domain rather than unrestricted prediction across novel scaffold space. Similarly, because the GA did not impose an explicit applicability-domain or uncertainty constraint, the predicted sub-8 nM values were used as internal selection criteria rather than prospective potency claims. This distinction is important because the experimentally measured HTRF activities, rather than the SVR outputs, provide the relevant biochemical evidence for the final compounds. Future iterations of the platform could further strengthen generalizability through scaffold-stratified or independent external validation, applicability-domain analysis, and uncertainty-aware molecular generation.

Both designed compounds were modeled within the BMS-202-associated interfacial region and retained interactions with residues implicated in small-molecule recognition (Figure 3b–d). PD-L1-Ser was predicted to combine aromatic contacts involving Tyr56 and Tyr123 with polar or ionic interactions around Ser117, Asp122, and Lys124. PD-L1-Ser-OEt occupied a similar region, indicating that esterification preserved the overall binding hypothesis while modifying the local physicochemical environment of the side chain. MM/PBSA calculations yielded mean trajectory-based energetic estimates of −114.287 ± 13.214 kJ/mol for PD-L1-Ser and −119.157 ± 14.887 kJ/mol for PD-L1-Ser-OEt (Table 3). Because the difference between these mean values was small relative to the associated trajectory variability, the calculation does not provide a quantitative basis for ranking their affinities. Instead, the stronger biochemical activity of PD-L1-Ser-OEt is established directly by the HTRF assay, in which its IC_50_ was approximately nine-fold lower than that of PD-L1-Ser. The potency difference may therefore reflect a combination of local packing, solvation, conformational behavior, and physicochemical properties that are not fully captured by the present simulations.

The cross-species structural analysis provides additional context for interpreting the murine efficacy experiment. Human and mouse PD-L1 retain closely related IgV-domain folds, yet several pocket-proximal differences, including Ile54/Val54, Asn63/Gln63, Arg113/Cys113, and Met115/Ile115, together with altered Tyr56 side-chain positioning, can influence the geometry and conformational adaptability of the small-molecule-binding region [22,25]. Docking to a 5J89-templated mouse PD-L1 dimer likewise produced ligand poses displaced from the canonical crystallographic human BMS-202 orientation. These findings provide a plausible structural basis for species-dependent ligand recognition. Accordingly, the human HTRF assay and the murine 4T1 experiment should be viewed as complementary but mechanistically distinct levels of evidence: the former demonstrates inhibition of the recombinant human PD-1/PD-L1 interaction, whereas the latter demonstrates antitumor pharmacodynamic activity in an immunocompetent mouse model. Establishing the contribution of direct murine PD-L1 engagement will require mouse-specific binding or functional checkpoint assays.

The experimental activity profile further helps distinguish the pharmacological characteristics of the two candidates. Both compounds displayed relatively limited direct growth inhibition in MDA-MB-231 and 4T1 monocultures compared with BMS-202 (Figure 5c,d; Table 5 and Table 6), indicating that strong direct tumor-cell cytotoxicity is unlikely to fully account for their in vivo effects. In contrast, treatment of immunocompetent 4T1-bearing mice produced pronounced reductions in tumor burden, particularly with PD-L1-Ser-OEt (Figure 6b–e; Table 7), while body weight remained broadly stable during the short treatment period (Figure 6f). Serum IFN-γ and IL-4 concentrations also increased in several candidate-treated groups, with the largest changes observed at the higher PD-L1-Ser-OEt doses (Figure 7a,b; Table 8). IFN-γ is a hallmark effector cytokine associated with cytotoxic T-cell activation and antitumor immunity, whereas IL-4 may reflect broader T-helper polarization and immune remodeling [26,27,28]. However, these circulating cytokines could be considered as secondary systemic pharmacodynamic readouts rather than direct markers of tumor-infiltrating T-cell activation. Nevertheless, together with the biochemical blockade and in vivo efficacy data, they are compatible with a treatment-associated immune component that warrants more direct cellular investigation.

Among the two candidates, PD-L1-Ser-OEt consistently showed the more favorable experimental profile, combining an HTRF IC_50_ of 0.2068 μM with the greatest reduction in tumor burden in the 4T1 study. Esterification of the serine carboxyl group provides a plausible medicinal-chemistry explanation for this improvement. Masking the carboxylate would be expected to reduce ionization and increase lipophilicity, which can favor membrane permeability and local tissue exposure [29,30,31]. In addition, PD-L1-Ser-OEt could potentially undergo esterase-mediated hydrolysis to PD-L1-Ser in vivo, providing a soft-prodrug-like relationship between the two analogues [32,33,34]. These possibilities remain mechanistic hypotheses rather than established pharmacokinetic mechanisms, but they provide a rational basis for prioritizing PD-L1-Ser-OEt for further optimization. Importantly, because the present efficacy study used intratumoral administration, future systemic exposure studies will be needed to determine whether this ester modification also improves pharmacokinetic behavior after clinically relevant routes of administration.

The present findings therefore position PD-L1-Ser and, in particular, PD-L1-Ser-OEt as early-stage lead scaffolds. Further development will focus on three areas: direct cellular and mouse-specific confirmation of checkpoint modulation and intratumoral immune responses; pharmacokinetic, systemic-dose, and tissue-distribution studies; and broader assessment of solubility, metabolic stability, selectivity, aggregation, and off-target activity. These studies will help define the relationship between the structural features identified here and the pharmacological performance of the scaffold, while providing a basis for subsequent medicinal-chemistry optimization.

## 4. Materials and Methods

### 4.1. Materials and Reagents

Human breast cancer MDA-MB-231 cells and mouse breast cancer 4T1 cells were purchased from the Type Culture Collection of the Chinese Academy of Sciences (Shanghai, China). Female BALB/c mice (6–8 weeks old, 16–20 g) were obtained from the Laboratory Animal Center of Jiangsu University (Zhenjiang, China). The reference compound BMS202 (CAS No. 1675203-84-5) and 5-fluorouracil (5-FU) were purchased from Shanghai Taoshu Biotechnology Co., Ltd. (Shanghai, China) and Bidepharm (Shanghai, China), respectively. Dimethyl sulfoxide (DMSO) and other routine reagents were obtained from Sinopharm Chemical Reagent Co., Ltd. (Shanghai, China). DMEM high-glucose medium, RPMI-1640 medium, fetal bovine serum (FBS), penicillin-streptomycin, trypsin, and phosphate-buffered saline (PBS) were purchased from Gibco (Thermo Fisher Scientific, Waltham, MA, USA) and BIOEXPLORER (Beijing, China). The homogeneous time-resolved fluorescence (HTRF) PD-1/PD-L1 binding assay kit was purchased from Cisbio (Codolet, France). The Cell Counting Kit-8 (CCK-8) was obtained from Sigma-Aldrich (Darmstadt, Germany). Mouse interferon-γ (IFN-γ) and interleukin-4 (IL-4) ELISA kits were purchased from Shanghai Yubo Biotechnology Co., Ltd. (Shanghai, China). All chemicals and solvents for chemical synthesis were obtained from commercial suppliers (Bidepharm, Shanghai, China; Saen Chemical, Shanghai, China; and Sinopharm Chemical Reagent Co., Ltd., Shanghai, China) and used without further purification.

### 4.2. Data Collection and Preprocessing

Small-molecule PD-1/PD-L1 inhibitors and their activity data were collected from public databases and the literature, including PubChem (https://pubchem.ncbi.nlm.nih.gov/, accessed on 12 January 2025), the RCSB Protein Data Bank (https://www.rcsb.org/, accessed on 12 January 2025), BindingDB (http://www.bindingdb.org/, accessed on 12 January 2025), and relevant patents and peer-reviewed articles. The RCSB Protein Data Bank was used to obtain protein-ligand structural templates. Chemical structures were standardized and converted to canonical SMILES with OpenBabel 2.4.1. Extended-connectivity fingerprints (ECFPs; radius = 2) were generated with RDKit v2020.09.1 to encode molecular structure. IC_50_ values (nmol·L-1) were transformed to their negative logarithmic form (pIC_50_) to improve numerical stability for regression modeling. The resulting dataset of 1385 compounds was randomly divided at a 4:1 ratio into a training set (*n* = 1108) and an internal hold-out test set (*n* = 277). Because this split was generated from the same curated dataset and was not scaffold-stratified, it was used for internal model assessment rather than independent external validation. No independent external dataset was evaluated in the present study.

### 4.3. SVR-GA Integrated Model for Activity Prediction and Molecular Generation

To enable de novo generation of novel PD-L1 inhibitor candidates with improved predicted potency, an integrated SVR-GA activity prediction and molecular design platform was established. The SVR model was implemented in scikit-learn (version 1.7.1) to predict the activity of PD-1/PD-L1 small-molecule inhibitors. ECFP fingerprints were used as input descriptors and pIC_50_ values as regression targets. The initial model employed a polynomial kernel. Key hyperparameters included the penalty parameter C (balancing model complexity against training error) and gamma (controlling the influence radius of training samples in kernel space). Three-fold cross-validation was used during hyperparameter tuning to reduce overfitting.

Model performance was evaluated using root mean square error (RMSE; Equation (1)) and the coefficient of determination (R^2^; Equation (2)). RMSE reflects the magnitude of prediction error in the original response scale, whereas R^2^ describes the proportion of variance explained by the model.

(1) $$RMSE=\sqrt{\frac{1}{m}\sum_{i=1}^{m}{\left(y_{i}-\hat{y_{i}}\right)}^{2}},$$

(2) $$R^{2}=1-\frac{\sum_{i=1}^{m}{\left(y_{i}-\hat{y_{i}}\right)}^{2}}{\sum_{i=1}^{m}{\left(y_{i}-\bar{y}\right)}^{2}},$$

To optimize predictive performance, grid search was performed over combinations of kernel functions and hyperparameters under different cross-validation settings (three-, five-, seven-, and ten-fold). The penalty parameter C was explored at 1.0 and 1 × 10^5^; kernel functions included sigmoid, linear, polynomial, and radial basis function (RBF); gamma values of 0.1, 0.01, 0.001, and 0.0001 were tested. RMSE and R^2^ were jointly considered to select the most suitable model.

Based on the SVR model, an automated design platform for PD-L1 small-molecule inhibitors was developed using a genetic algorithm (GA) framework. The SVR model served as the fitness function to prioritize candidates with improved predicted potency. The workflow comprised population initialization, fragment extraction, crossover-based offspring generation, fitness evaluation, and iterative molecular evolution. Briefly, the top 100 most active compounds in the dataset were selected as the initial population and converted to SMILES with OpenBabel 2.4.1. Molecular fragments were extracted with RDKit by identifying cleavable rotatable single bonds and corresponding connection sites. Random crossover at predefined attachment positions was performed with rdChemReactions to generate offspring molecules. Candidates with predicted IC_50_ values below 8 nM were retained as parents for the next generation. Evolution was continued for 1000 generations with a mutation probability of 0.01 to enrich structures with improved predicted activity.

During GA evolution, no explicit applicability-domain, nearest-neighbor similarity, scaffold-distance, or predictive-uncertainty constraint was imposed on generated candidates. Therefore, as molecular evolution proceeded, some candidates could enter regions of chemical space that were weakly represented by the training data, where SVR predictions would be increasingly extrapolative. Accordingly, the predicted IC_50_ < 8 nM criterion was used solely as an internal fitness and selection threshold. Generated compounds were not considered experimentally validated hits on the basis of the SVR prediction alone; final activity assessment was based on the measured HTRF inhibition data.

### 4.4. Molecular Dynamics-Based Identification of PD-L1 Binding Hot Spots

To characterize the druggable region at the interface of the PD-L1 homodimer (the dimeric form of PD-L1 that is targeted by small-molecule inhibitors) and to identify key interaction residues for subsequent structure-based screening, molecular dynamics (MD) simulations were performed on the PD-L1 homodimer in complex with a small-molecule ligand.

BMS202, a well-characterized small-molecule PD-L1 inhibitor that binds at the dimer interface and stabilizes the PD-L1 homodimer, was selected as a reference ligand because its co-crystal structure with PD-L1 (PDB ID: 5J89) has high resolution and its inhibitory activity has been extensively reported [12]. MD simulations were then performed for the PD-L1 dimer-BMS202 complex to investigate binding stability and pocket dynamics. Key interaction residues were identified through analysis of root mean square deviation (RMSD; Equation (3)), root mean square fluctuation (RMSF; Equation (4)), hydrogen bonding, and molecular mechanics/Poisson-Boltzmann surface area (MM/PBSA) binding free-energy calculations. Simulations were run for 100 ns in GROMACS 2020 using the amber99sb-ildn force field. Ligand parameters and RESP charges were generated with Antechamber (AmberTools25) and ORCA (version 6.1) and converted to GROMACS-compatible formats. The protein-ligand complex was solvated in a periodic water box, neutralized with counterions, energy-minimized by steepest descent (maximum 50,000 steps), and equilibrated under NVT and NPT conditions for approximately 1 ns in total. Long-range electrostatics were treated using the particle mesh Ewald method with a 1.0 nm cutoff. The production run was carried out with a 2 fs time step, and frames were saved every 0.2 ps.

After simulation, trajectories were analyzed for RMSD, RMSF, hydrogen bonds, and MM/PBSA binding free energy using g_mmpbsa (version 3.0.0). Hydrogen-bond occupancies were further evaluated with VMD 1.9.3.

(3) $$RMSD(t)=\sqrt{\frac{1}{N}\sum_{i=1}^{N}{\left\|r_{i}\left(t\right)-r_{i}^{ref}\right\|}^{2}},$$

(4) $${RMSF}_{i}=\sqrt{\frac{1}{T}\sum_{t=1}^{T}{\left\|r_{i}\left(t\right)-\langle r_{i}\rangle \right\|}^{2}},$$

### 4.5. Flexible Docking of Candidate Compounds to PD-L1

To prioritize the GA-generated candidates for experimental validation, flexible docking was performed against the PD-L1 dimer structure (PDB ID: 5J89). Candidate molecules were energy-minimized and prepared using AutoDock Tools 1.5.6 by protonation, charge assignment, and definition of rotatable bonds. The PD-L1 dimer was prepared by removing the co-crystallized ligand and water molecules, followed by hydrogen addition and charge assignment. Flexible residues and the docking grid were defined on the basis of the MD-identified interfacial pocket. AutoDock Vina (version 1.2.7) was used for docking.

BMS-202 was first redocked into its crystallographic binding site as a pose-recovery control. An RMSD of <2 Å between the redocked and crystallographic poses was considered indicative of successful reproduction of the experimentally observed binding orientation.

To further assess stochastic reproducibility, BMS-202, BMS-1001, BMS-1166, PD-L1-Ser, and PD-L1-Ser-OEt were each subjected to three independent docking runs using random seeds of 101, 202, and 303. The receptor preparation, docking box, flexible-residue definition, exhaustiveness, and all other docking parameters were kept identical among runs. The best-ranked Vina score from each run was recorded, and docking-score reproducibility was summarized as mean ± SD. Top-ranked poses were also compared for consistency of binding orientation and pocket interactions. Candidates were prioritized by considering docking score together with pocket occupancy, engagement of key interfacial residues, interaction patterns, and synthetic feasibility.

### 4.6. MD Simulation of Candidate Compounds Binding to PD-L1

To further assess binding stability, 100 ns MD simulations were performed for the PD-L1 complexes of the prioritized candidates using the docked poses as initial conformations. System stability was evaluated by RMSD analysis, and MM/PBSA calculations were used to estimate binding free energies and their components, including van der Waals, electrostatic, polar solvation, and nonpolar solvation terms.

### 4.7. Synthesis of Candidate Compounds

All reagents and solvents were purchased from commercial suppliers and used as received unless otherwise noted. Reactions were monitored by thin-layer chromatography (TLC) on silica gel plates, and column chromatography was performed on silica gel (200–300 mesh). High-resolution mass spectra (HRMS) were recorded on a Q Exactive Plus ESI-TOF mass spectrometer (Thermo Fisher Scientific, Waltham, MA, USA) in positive ion mode. ^1^H NMR and ^13^C NMR spectra were recorded on a 400 MHz spectrometer (UltraShield 400, Bruker BioSpin, Billerica, MA, USA) using DMSO-d6 as solvent and tetramethylsilane (TMS) as internal standard. Chemical shifts (δ) are reported in parts per million (ppm) and coupling constants (J) in Hz.

The synthetic route was established via retrosynthetic disconnection. The two ether linkages were disconnected to give 2,4-dihydroxybenzaldehyde as the core starting material. Electrophilic chlorination introduced chlorine at the 5-position. For the pyridine side chains, a bromomethyl intermediate was prepared by Appel-type bromination of 5-(hydroxymethyl) nicotinonitrile. Williamson etherification connected the two fragments, followed by reductive amination with L-serine or its ethyl ester to afford the target compounds.

#### 4.7.1. Synthesis of Intermediate 1: 5-Chloro-2,4-dihydroxybenzaldehyde

2,4-Dihydroxybenzaldehyde (2.00 g, 14.40 mmol) was dissolved in chloroform (30 mL), and N-chlorosuccinimide (1.9 g, 14.40 mmol) was added. The mixture was stirred at 60 °C for 1 h. Concentrated HCl (260 μL) was added dropwise with caution, and stirring continued at 60 °C for 8 h. After cooling, the mixture was extracted with brine and ethyl acetate. The combined organic layers were dried, filtered, and concentrated. The residue was purified by column chromatography (petroleum ether/ethyl acetate = 3:1) to afford the product as a solid. ^1^H NMR (400 MHz, DMSO-d6) δ 11.64 (s, 1H), 10.79 (s, 1H), 9.78 (s, 1H), 7.52 (s, 1H), 6.57 (s, 1H).

#### 4.7.2. Synthesis of Intermediate 2: 5-(Bromomethyl)nicotinonitrile

5-(Hydroxymethyl)nicotinonitrile (4.0 g, 29.8 mmol) and triphenylphosphine (9.4 g, 35.8 mmol) were dissolved in dichloromethane (30 mL). N-Bromosuccinimide (6.4 g, 35.8 mmol) was added, turning the mixture light brown. After purging with nitrogen, the mixture was stirred at 28 °C for 12 h. The crude product was purified by column chromatography (petroleum ether/ethyl acetate, gradient 100:1 to 3:1) and concentrated to give a yellow oil. ^1^H NMR (400 MHz, CDCl3) δ 8.90 (d, J = 2.0 Hz, 1H), 8.85 (d, J = 2.0 Hz, 1H), 7.99 (t, J = 2.0 Hz, 1H), 4.58 (s, 2H).

#### 4.7.3. Synthesis of Intermediate 3: 5,5′-(((4-Chloro-6-formyl-1,3-phenylene)bis(oxy))bis(methylene))dinicotinonitrile

5-(Bromomethyl)nicotinonitrile (3.2 g, 16.3 mmol) was dissolved in N,N-Dimethylformamide (DMF, 50 mL). Cesium carbonate (4.4 g, 13.6 mmol) was added, and the mixture was stirred at room temperature for 20 min. Then 5-chloro-2,4-dihydroxybenzaldehyde (936 mg, 5.4 mmol) was added, and the reaction was stirred at 80 °C for 2 h. The solvent was removed, and the residue was triturated with petroleum ether (20 mL) and filtered to give a yellow-brown solid. Due to instability during chromatography, the product was used without further purification. HRMS (ESI+) *m*/*z* [M+H]^+^ calcd for C_21_H_14_ClN_4_O_3_ 405.0754, found 405.0758.

#### 4.7.4. Synthesis of the Candidate Compounds

The above dialdehyde (100 mg, 0.25 mmol) was dissolved in DMF (35 mL). A solution of the appropriate amine (L-serine or DL-serine ethyl ester hydrochloride, 130 mg, 1.24 mmol) in water (5 mL) was added, followed by N,N-Diisopropylethylamine (DIPEA, 0.083 mL, 0.5 mmol). The mixture was stirred at 55 °C for 4 h. After cooling to room temperature, the pH was adjusted to 6 with trifluoroacetic acid (TFA). Sodium cyanoborohydride (47 mg, 0.75 mmol) was added slowly, and stirring continued at room temperature for 4 h. The solvent was removed, and the product was purified by column chromatography (CH_2_Cl_2_/MeOH = 10:1).

For the compound from L-serine: HRMS (ESI+) *m*/*z* [M+H]^+^ calcd for C_23_H_22_N_5_O_6_ 494.1231, found 494.1214. ^1^H NMR (400 MHz, DMSO-d6) δ 9.04 (s, 1H), 9.00 (s, 1H), 8.95 (s, 1H), 8.45 (s, 1H), 8.38 (s, 1H), 7.62 (s, 1H), 7.12 (s, 1H), 5.43 (s, 2H), 5.33 (s, 2H), 4.22 (m, 2H), 3.93 (s, 1H), 3.88 (m, 2H). ^13^C NMR (100 MHz, DMSO-d6) δ 169.15, 158.71, 158.44, 156.99, 155.14, 152.77, 152.59, 139.37, 139.23, 133.61, 133.05, 132.98, 117.36, 117.22, 114.17, 113.67, 109.53, 109.51, 100.65, 67.81, 67.60, 60.85, 59.17, 43.61.

For the compound from DL-serine ethyl ester hydrochloride: HRMS (ESI+) m/z [M+H]^+^ calcd for C_25_H_26_N_5_O_6_ 522.1524, found 522.1609. ^1^H NMR (400 MHz, DMSO-d6) δ 9.03 (s, 1H), 8.98 (s, 1H), 8.94 (s, 1H), 8.50 (s, 1H), 8.38 (s, 1H), 7.61 (s, 1H), 7.13 (s, 1H), 5.40 (s, 2H), 5.34 (s, 2H), 4.22 (s, 2H), 4.15 (m, 1H), 4.10 (m, 2H), 3.87 (m, 2H), 1.20 (t, 3H). ^13^C NMR (100 MHz, DMSO-d6) δ 167.73, 158.39, 157.07, 155.20, 152.72, 152.69, 152.60, 152.47, 139.46, 139.24, 133.75, 133.05, 117.34, 117.22, 114.01, 113.68, 109.53, 109.52, 100.68, 67.81, 67.62, 62.45, 60.81, 59.37, 43.72, 40.10, 14.29.

### 4.8. HTRF-Based In Vitro Assessment of PD-1/PD-L1 Blockade

To evaluate the inhibitory effects of the candidate compounds on the PD-1/PD-L1 interaction, a homogeneous time-resolved fluorescence (HTRF) assay was performed.

The HTRF Human PD-1/PD-L1 assay contained recombinant human Tag1-PD-L1 (25 nM), recombinant human Tag2-PD-1 (250 nM), anti-Tag1-Eu donor reagent (100-fold dilution), and anti-Tag2-XL665 acceptor reagent (25-fold dilution). BMS202 was used as a positive control and diluted to 1 μM for the assay. Candidate compounds PD-L1-Ser and PD-L1-Ser-OEt were prepared as DMSO stock solutions and serially diluted to the desired working concentrations (0.1–1000 μM; Table 4). The assay was performed in 96-well plates with different experimental groups, including control and compound-treated groups, each in triplicate, with a final volume of 20 μL per well. After incubation at room temperature for 15 min, 10 μL of the donor/acceptor probe mixture was added to each well, followed by incubation for 2 h in the dark. Fluorescence was measured at an excitation wavelength of 320 nm and emission wavelengths of 620 nm and 665 nm using a multimode plate reader (Synergy HTX, BioTek Instruments, Winooski, VT, USA). The emission ratio (ER) and binding rate were calculated using Equations (5) and (6).

(5) $$ER=\frac{{Emission\;Signal}_{665nm}}{{Emission\;Signal}_{620nm}},$$

(6) $$Binding\left(\%\right)=\frac{{ER}_{sample}-{ER}_{negative\;control}}{{ER}_{positive\;control}-{ER}_{negative\;control}}\times 100\%,$$

All data are presented as mean ± standard deviation (SD).

### 4.9. In Vitro Evaluation of Antitumor Activity of the Candidate Compounds

Human breast cancer MDA-MB-231 cells and murine 4T1 breast cancer cells were used to evaluate the in vitro antitumor effects of PD-L1-Ser and PD-L1-Ser-OEt. Nine groups were included: high-, medium-, and low-dose groups for each compound, a BMS202 positive-control group, a 0.1% Dimethyl sulfoxide (DMSO) negative-control group, and a blank-control group. Cells in the logarithmic growth phase were seeded in 96-well plates, allowed to attach for 24 h, and then treated with the indicated compounds or controls for 48 h. Cell viability was assessed using the Cell Counting Kit-8 (CCK-8) assay by measuring absorbance at 450 nm with a microplate reader (ELx800, BioTek Instruments, Winooski, VT, USA), and viability was calculated according to Equation (7).

Data were analyzed in GraphPad Prism and are presented as mean ± SD. IC_50_ values for BMS202, PD-L1-Ser, and PD-L1-Ser-OEt in MDA-MB-231 and 4T1 cells were calculated in GraphPad Prism.

(7) $$Cell\;viability\left(\%\right)=\frac{{OD}_{treatment}-{OD}_{blank}}{{OD}_{control}-{OD}_{blank}}\times 100\%,$$

### 4.10. Animals and Treatment

To evaluate the antitumor effects of PD-L1-Ser and its esterified derivative PD-L1-Ser-OEt in an intact immune system, a syngeneic 4T1 breast tumor model was established in female BALB/c mice. All animal experimental procedures were performed in accordance with the Guidelines for the Care and Use of Laboratory Animals formulated by the National Institutes of Health, and the protocols were approved by the Ethics Committee of Jiangsu University (Approval No. UJS-IACUC-2021032602). The individual mouse was considered the experimental unit.

A total of 45 female BALB/c mice (SPF grade, 6–8 weeks old) were obtained from the Laboratory Animal Centre of Jiangsu University and acclimatized for 1 week in an SPF facility. Mice were housed five per standard polycarbonate cage at 25 ± 1 °C and 70 ± 5% relative humidity under a 12 h light/12 h dark cycle, with ad libitum access to standard laboratory chow and sterile water.

Forty mice were inoculated subcutaneously in the left axillary region with 1 × 10^7^ 4T1 cells per mouse; the remaining five mice served as non-tumor-bearing healthy controls. Tumor-bearing mice were eligible for randomization when individual tumor volumes reached approximately 800 mm^3^. The 40 tumor-bearing mice were stratified by baseline tumor volume and allocated by block randomization to eight groups (*n* = 5 per group): vehicle control; 5-fluorouracil (5-FU, 25 mg/kg); PD-L1-Ser at 5, 25, or 50 mg/kg; and PD-L1-Ser-OEt at 5, 25, or 50 mg/kg. The healthy blank control group comprised five non-tumor-bearing mice receiving saline. 5-FU (25 mg/kg) was included as a conventional cytotoxic reference treatment to provide a pharmacodynamic benchmark for tumor-growth suppression in the 4T1 model. It was not intended as a mechanistic or target-specific control for PD-L1 inhibition. Because 5-FU was administered intravenously whereas PD-L1-Ser and PD-L1-Ser-OEt were administered intratumorally, comparisons between 5-FU and the candidate compounds were considered as contextual efficacy comparisons rather than direct target-specific comparisons. The sample size was selected on the basis of previous comparable studies and preliminary observations; no formal a priori power calculation was performed. No additional exclusion criteria were prespecified.

PD-L1-Ser and PD-L1-Ser-OEt were formulated in saline containing 2% DMSO and 40% PEG 300 and administered intratumorally at 50 μL per mouse once daily for 7 consecutive days. The vehicle-control group received 50 μL of the same vehicle by the same route and schedule. 5-FU was prepared in saline and administered via the tail vein at 25 mg/kg during the 7-day treatment period. Intratumoral and intravenous injections were performed under inhalational anesthesia with 1.5–2.0% isoflurane in oxygen delivered via a nose cone.

Block randomization was performed by an investigator who was not involved in tumor measurement or data analysis. Intratumoral treatment formulations were coded so that the personnel administering them were unaware of compound identity and dose. Because 5-FU was administered intravenously whereas the candidate compounds were administered intratumorally, complete blinding of treatment administration across all groups was not feasible. Tumor measurements and data analysis were performed by investigators blinded to group identity.

Mice were observed at least once daily for general condition and behavior, and body weight was recorded daily throughout treatment. Tumor length and width were measured every other day using digital calipers, and tumor volume was calculated as (width^2^ × length)/2. The primary efficacy outcome was longitudinal tumor volume; secondary outcomes were terminal tumor weight, tumor inhibition rate, tumor-free body weight, and serum IFN-γ and IL-4 concentrations. Tumor inhibition rate (%) was calculated as [1 − (mean tumor weight of the treated group/mean tumor weight of the vehicle-control group)] × 100. Tumor burden was maintained below 10% of body weight. Prespecified humane endpoints were tumor volume > 2500 mm^3^ or body-weight loss > 20%; animals meeting either endpoint were to be euthanized immediately. At study completion, terminal blood samples were collected for serum cytokine analysis, mice were euthanized by carbon dioxide inhalation in accordance with the approved institutional protocol, and tumors were excised and weighed. No animals or data points were excluded from the analysis.

### 4.11. Serum IFN-γ and IL-4 Detection by ELISA

The serum levels of IFN-γ and IL-4 were measured using Enzyme-linked immunosorbent assay (ELISA) kits according to the manufacturers’ instructions. Briefly, samples and HRP-conjugated detection antibodies were added to antibody-precoated plates to form sandwich complexes. After washing, TMB substrate was added, the reaction was stopped, and absorbance was read at 450 nm. Standard curves were prepared using serial dilutions of reference standards (IFN-γ: 50–1600 ng/L; IL-4: 15–480 pg/mL). Serum samples were diluted fivefold and assayed in triplicate. Plates were incubated at 37 °C for 30 min, washed five times, incubated with enzyme reagent for an additional 30 min, washed again, and then developed with substrate for 15 min before addition of stop solution. Concentrations were interpolated from standard curves and corrected for the dilution factor (Supplementary Materials, Figures S1 and S2).

### 4.12. Data Analysis

Statistical analyses were performed using GraphPad Prism version 8.01 (GraphPad Software, San Diego, CA, USA). Data are presented as mean ± SD unless otherwise stated. For the in vivo experiments, the individual mouse was considered the experimental unit. Longitudinal tumor-volume and body-weight measurements were analyzed using two-way repeated-measures ANOVA with treatment and time as factors, followed by Dunnett-adjusted multiple comparisons between each treatment group and the vehicle-treated tumor-bearing group. Terminal tumor weights were analyzed using one-way ANOVA followed by Dunnett’s multiple-comparisons test against the vehicle-treated tumor-bearing group. Serum IFN-γ and IL-4 concentrations were analyzed using one-way ANOVA followed by Dunnett’s multiple-comparisons against the saline-treated tumor-bearing group. The non-tumor-bearing healthy group was included as a physiological reference. Concentration-response curves for the HTRF and CCK-8 assays were fitted using four-parameter logistic nonlinear regression, from which IC_50_ values were estimated. Tumor-inhibition rates were calculated from terminal tumor-weight data as described in Section 4.10 and were reported as derived descriptive measures; statistical comparisons were performed using the underlying individual tumor-weight measurements. All tests were two-sided, and *p* < 0.05 was considered statistically significant.

## 5. Conclusions

In summary, we present an end-to-end platform integrating machine learning, molecular evolution, and structure-based simulation for the discovery of small-molecule PD-L1 inhibitors with experimental validation. This platform led to the identification of two candidates, PD-L1-Ser and PD-L1-Ser-OEt, which disrupted PD-1/PD-L1 binding in an HTRF assay and suppressed tumor growth in an immunocompetent 4T1 model without obvious body-weight loss, with PD-L1-Ser-OEt showing consistently stronger efficacy. This work highlights a scalable computational-to-experimental pipeline for immune checkpoint drug discovery. Further studies focusing on cell-based checkpoint blockade assays, intratumoral immune profiling, pharmacokinetics, and safety evaluation will be essential for clarifying mechanism and supporting lead optimization toward translational development.

## Figures and Tables

**Table 1:** Hydrogen-bond interactions between BMS202 and the PD-L1 dimer.

Donor	Acceptor	Occupancy
BMS202-N1	ASP122-O1	63.14%
LYS124-N1	BMS202-O1	62.49%

**Table 2:** MM/PBSA binding free-energy components of BMS202 with the PD-L1 dimer.

Compound	Δ_VDW_	Δ_Elec_	Δ_Apol_	Δ_Pol_	Δ_E_
BMS202	−261.960 ± 11.120	−42.842 ± 8.676	−21.666 ± 0.897	159.158 ± 10.786	−167.310 ± 11.913

Notes: Δ_VDW_, van der Waals energy; Δ_Elec_, electrostatic energy; Δ_Apol_, nonpolar solvation energy; Δ_Pol_, polar solvation energy; Δ_E_, total binding free energy (kJ/mol).

**Table 3:** MM/PBSA binding free-energy components of the candidate compounds with the PD-L1 dimer.

Compound	Δ_VDW_	Δ_Elec_	Δ_Apol_	Δ_Pol_	Δ_E_
PD-L1-Ser	−245.268 ± 11.806	−129.174 ± 23.077	−21.085 ± 0.729	281.240 ± 24.073	−114.287 ± 13.214
PD-L1-Ser-OEt	−261.821 ± 14.819	−123.502 ± 15.588	−23.646 ± 0.981	289.812 ± 18.224	−119.157 ± 14.887

Notes: Δ_VDW_, van der Waals energy; Δ_Elec_, electrostatic energy; Δ_Apol_, nonpolar solvation energy; Δ_Pol_, polar solvation energy; Δ_E_, total binding free energy (kJ/mol).

**Table 4:** Inhibition of PD-1/PD-L1 binding by PD-L1-Ser and PD-L1-Ser-OEt in the HTRF assay.

Candidates	IC_50_ (μM)
PD-L1-Ser	1.8520
PD-L1-Ser-OEt	0.2068

**Table 5:** Cell viability of MDA-MB-231 cells after treatment with candidate compounds.

Group	IC_50_ (µg/mL)
PD-L1-Ser	402.2
PD-L1-Ser-OEt	338.5
BMS202	47.68

**Table 6:** Cell viability of 4T1 cells after treatment with candidate compounds.

Group	IC_50_ (µg/mL)
PD-L1-Ser	303.6
PD-L1-Ser-OEt	174.4
BMS202	27.78

**Table 7:** In vivo antitumor efficacy of PD-L1-Ser and PD-L1-Ser-OEt in the 4T1 model. (mean ± SD, *n* = 5).

Group	Tumor Volume (cm^3^)	Tumor Weight (g)	Inhibition Rate (%)
Saline	2.46 ± 0.35	2.26 ± 0.38	/
5-FU (25 mg/kg)	0.89 ± 0.05	1.00 ± 0.53	55.75%
PD-L1-Ser (5 mg/kg)	1.53 ± 0.11	1.59 ± 0.29	29.47%
PD-L1-Ser (25 mg/kg)	1.25 ± 0.17	1.38 ± 0.60	38.94%
PD-L1-Ser (50 mg/kg)	0.95 ± 0.19	0.98 ± 0.52	56.64%
PD-L1-Ser-OEt (5 mg/kg)	0.35 ± 0.25	0.38 ± 0.17	83.27%
PD-L1-Ser-OEt (25 mg/kg)	0.24 ± 0.17	0.31 ± 0.20	86.46%
PD-L1-Ser-OEt (50 mg/kg)	0.15 ± 0.05	0.14 ± 0.05	93.81%

**Table 8:** Serum IFN-γ and IL-4 levels in 4T1 tumor-bearing mice. (mean ± SD, *n* = 5).

Group	IFN-γ (ng/L)	IL-4 (pg/mL)
Saline	296.53 ± 18.20	93.78 ± 21.95
5-FU (25 mg/kg)	302.32 ± 22.07	91.50 ± 9.51
PD-L1-Ser (5 mg/kg)	304.49 ± 36.49	100.37 ± 13.87
PD-L1-Ser (25 mg/kg)	318.23 ± 21.50	104.68 ± 7.83
PD-L1-Ser (50 mg/kg)	381.91 ± 29.33	145.48 ± 12.70
PD-L1-Ser-OEt (5 mg/kg)	391.32 ± 16.85	136.61 ± 51.52
PD-L1-Ser-OEt (25 mg/kg)	453.55 ± 20.47	188.56 ± 30.46
PD-L1-Ser-OEt (50 mg/kg)	563.89 ± 43.49	253.18 ± 14.34
Healthy control	297.25 ± 12.82	101.89 ± 7.19

## Data Availability

The original contributions presented in this study are included in the article/Supplementary Material. Further inquiries can be directed to the corresponding author.

## Supplementary Materials

The following supporting information can be downloaded at: https://www.mdpi.com/article/10.3390/ph19091439/s1, Figure S1: Chemical structures and reported HTRF inhibitory activities of representative BMS-series human PD-L1 inhibitors. Figure S2: MD analysis of the PD-L1 dimer bound to BMS202. Figure S3. Structural comparison of the proposed small-molecule binding region between human and mouse PD-L1. Figure S4. Docking results of BMS-202, PD-L1-Ser, and PD-L1-Ser-OEt to a 5J89-templated mouse PD-L1 dimer model. Figure S5. Structural characterization of PD-L1-Ser and PD-L1-Ser-OEt. Table S1. Reproducibility of AutoDock Vina docking scores across three independent random seeds.

## Abbreviations

The following abbreviations are used in this manuscript:

PD-L1	Programmed Death-Ligand 1
PD-1	Programmed Death-1
SVR	Support Vector Regression
GA	Genetic Algorithm
MD	Molecular Dynamics
HTRF	Homogeneous Time-Resolved Fluorescence
IC_50_	Half-Maximal Inhibitory Concentration
pIC_50_	The Negative Logarithm of IC_50_
RMSE	Root Mean Square Error
RMSD	Root Mean Square Deviation
RMSF	Root Mean Square Fluctuation
MM/PBSA	Molecular Mechanics/Poisson-Boltzmann Surface Area
TLC	Thin-Layer Chromatography
HRMS	High-Resolution Mass Spectrometry
NMR	Nuclear Magnetic Resonance
ELISA	Enzyme-Linked Immunosorbent Assay
ECFP	Extended-Connectivity Fingerprint
DMSO	Dimethyl Sulfoxide
DMF	N,N-Dimethylformamide
DIPEA	N,N-Diisopropylethylamine
TFA	Trifluoroacetic Acid
TMS	Tetramethylsilane
5-FU	5-Fluorouracil

## References

[1] Ai L., Xu A., Xu J., Xu J. (2020). Roles of PD-1/PD-L1 Pathway: Signaling, Cancer, and Beyond. Regulation of Cancer Immune Checkpoints.

[2] Parvez A., Choudhary F., Mudgal P., Khan R., Qureshi K.A., Farooqi H., Aspatwar A. (2023). PD-1 and PD-L1: Architects of immune symphony and immunotherapy breakthroughs in cancer treatment. Front. Immunol..

[3] Shi L., Chen S., Yang L., Li Y. (2013). The role of PD-1 and PD-L1 in T-cell immune suppression in patients with hematological malignancies. J. Hematol. Oncol..

[4] Wei F., Zhong S., Ma Z., Kong H., Medvec A., Ahmed R., Freeman G.J., Krogsgaard M., Riley J.L. (2013). Strength of PD-1 signaling differentially affects T-cell effector functions. Proc. Natl. Acad. Sci. USA.

[5] Wu M., Huang Q., Xie Y., Wu X., Ma H., Zhang Y., Xia Y. (2022). Improvement of the anticancer efficacy of PD-1/PD-L1 blockade via combination therapy and PD-L1 regulation. J. Hematol. Oncol..

[6] Javed S.A., Najmi A., Ahsan W., Zoghebi K. (2024). Targeting PD-1/PD-L-1 immune checkpoint inhibition for cancer immunotherapy: Success and challenges. Front. Immunol..

[7] Zhang F., Ramar S., Wang Y., Xu H., Zhang K., Awadasseid A., Rao G., Zhang W. (2025). Advances in cancer immunotherapy using small-molecular inhibitors targeting the PD-1/PD-L1 interaction. Bioorg. Med. Chem..

[8] Deng J., Cheng Z., Long J., Dömling A., Tortorella M., Wang Y. (2022). Small Molecule Inhibitors of Programmed Cell Death Ligand 1 (PD-L1): A Patent Review (2019–2021). Expert Opin. Ther. Pat..

[9] Sasmal P., Prabitha P., Prashantha Kumar B.R., Swetha B.R., Babasahib S.K., Raghavendra N.M. (2025). Beyond peptides: Unveiling the design strategies, structure activity correlations and protein-ligand interactions of small molecule inhibitors against PD-1/PD-L1. Bioorg. Chem..

[10] Liu C., Seeram N.P., Ma H. (2021). Small molecule inhibitors against PD-1/PD-L1 immune checkpoints and current methodologies for their development: A review. Cancer Cell Int..

[11] Zhang H., Xia Y., Yu C., Du H., Liu J., Li H., Huang S., Zhu Q., Xu Y., Zou Y. (2021). Discovery of Novel Small-Molecule Inhibitors of PD-1/PD-L1 Interaction via Structural Simplification Strategy. Molecules.

[12] Zak K.M., Grudnik P., Guzik K., Zieba B.J., Musielak B., Dömling A., Dubin G., Holak T.A. (2016). Structural basis for small molecule targeting of the programmed death ligand 1 (PD-L1). Oncotarget.

[13] Ganesan A., Ahmed M., Okoye I., Arutyunova E., Babu D., Turnbull W.L., Kundu J.K., Shields J., Agopsowicz K.C., Xu L. (2019). Comprehensive in vitro characterization of PD-L1 small molecule inhibitors. Sci. Rep..

[14] Guzik K., Zak K.M., Grudnik P., Magiera K., Musielak B., Törner R., Skalniak L., Dömling A., Dubin G., Holak T.A. (2017). Small-Molecule Inhibitors of the Programmed Cell Death-1/Programmed Death-Ligand 1 (PD-1/PD-L1) Interaction via Transiently Induced Protein States and Dimerization of PD-L1. J. Med. Chem..

[15] Awadasseid A., Wu M., Zhang F., Song Y., Wu Y., Zhang W. (2025). Novel PD-L1 Small-Molecule Inhibitors Advancing Cancer Immunotherapy. Anti-Cancer Agents Med. Chem..

[16] Chandrasekaran J., Elumalai S., Murugesan V., Kunjiappan S., Pavadai P., Theivendren P. (2023). Computational design of PD-L1 small molecule inhibitors for cancer therapy. Mol. Divers..

[17] Shi Y. (2021). Support vector regression-based QSAR models for prediction of antioxidant activity of phenolic compounds. Sci. Rep..

[18] Wang Y., Zheng M., Xiao J., Lu Y., Wang F., Lu J., Luo X., Zhu W., Jiang H., Chen K. (2010). Using support vector regression coupled with the genetic algorithm for predicting acute toxicity to the fathead minnow. SAR QSAR Environ. Res..

[19] McKearnan S.B., Vock D.M., Marai G.E., Canahuate G., Fuller C.D., Wolfson J. (2023). Feature selection for support vector regression using a genetic algorithm. Biostatistics.

[20] Deng J., Yang Z., Ojima I., Samaras D., Wang F. (2022). Artificial intelligence in drug discovery: Applications and techniques. Brief. Bioinform..

[21] Xu X., Luo S., Zhao X., Tang B., Zhang E., Liu J., Duan L. (2024). Computational analysis of PD-L1 dimerization mechanism induced by small molecules and potential dynamical properties. Int. J. Biol. Macromol..

[22] Magiera-Mularz K., Kocik J., Musielak B., Plewka J., Sala D., Machula M., Grudnik P., Hajduk M., Czepiel M., Siedlar M. (2021). Human and mouse PD-L1: Similar molecular structure, but different druggability profiles. iScience.

[23] Xu J., Kong Y., Zhu P., Du M., Liang X., Tong Y., Li X., Dong C. (2024). Progress in small-molecule inhibitors targeting PD-L1. RSC Med. Chem..

[24] Guo Y., Jin Y., Wang B., Liu B. (2021). Molecular Mechanism of Small-Molecule Inhibitors in Blocking the PD-1/PD-L1 Pathway through PD-L1 Dimerization. Int. J. Mol. Sci..

[25] Koblish H.K., Wu L., Wang L.-C.S., Liu P.C.C., Wynn R., Rios-Doria J., Spitz S., Liu H., Volgina A., Zolotarjova N. (2022). Characterization of INCB086550: A Potent and Novel Small-Molecule PD-L1 Inhibitor. Cancer Discov..

[26] Gocher A.M., Workman C.J., Vignali D.A.A. (2022). Interferon-γ: Teammate or opponent in the tumour microenvironment?. Nat. Rev. Immunol..

[27] Gao S., Hsu T.-W., Li M.O. (2021). Immunity beyond cancer cells: Perspective from tumor tissue. Trends Cancer.

[28] Dagher O.K., Schwab R.D., Brookens S.K., Posey A.D. (2023). Advances in cancer immunotherapies. Cell.

[29] Beaumont K., Webster R., Gardner I., Dack K. (2003). Design of Ester Prodrugs to Enhance Oral Absorption of Poorly Permeable Compounds: Challenges to the Discovery Scientist. Curr. Drug Metab..

[30] Thiele N.A., Sloan K.B. (2016). A Double Prodrug with Improved Membrane Permeability over the Parent Chelator HBED Provides Superior Cytoprotection against Hydrogen Peroxide. ChemMedChem.

[31] Jana S., Mandlekar S., Marathe P. (2010). Prodrug Design to Improve Pharmacokinetic and Drug Delivery Properties: Challenges to the Discovery Scientists. Curr. Med. Chem..

[32] Wang Y., Liu X., Zou X., Wang S., Luo L., Liu Y., Dong K., Yao X., Li Y., Chen X. (2021). Metabolism and Interspecies Variation of IMMH-010, a Programmed Cell Death Ligand 1 Inhibitor Prodrug. Pharmaceutics.

[33] Shi L., Wu X., Li T., Wu Y., Song L., Zhang W., Yin L., Wu Y., Han W., Yang Y. (2022). An esterase-activatable prodrug formulated liposome strategy: Potentiating the anticancer therapeutic efficacy and drug safety. Nanoscale Adv..

[34] Lai F., Ji M., Huang L., Wang Y., Xue N., Du T., Dong K., Yao X., Jin J., Feng Z. (2022). YPD-30, a prodrug of YPD-29B, is an oral small-molecule inhibitor targeting PD-L1 for the treatment of human cancer. Acta Pharm. Sin. B.

