# Supplementary material for: Integrated Machine Learning and Molecular Simulation-Guided Discovery of Novel Small-Molecule PD-L1 Inhibitors
Source: Pharmaceuticals (Basel). 2026 Sep 11;19(9):1439. doi: 10.3390/ph19091439 (PMC13610596; doi:10.3390/ph19091439)
Supplement: Supplementary file 1 [file pharmaceuticals-19-01439-s001.zip › pharmaceuticals-4515150-supplementary.pdf]

Supplementary file

1. Methods

To examine the cross-species relevance of the proposed human PD-L1-binding pocket, the amino acid sequences and experimentally determined IgV-domain structures of human PD-L1 and mouse PD-L1 were compared. Chain C of the human PD-L1 structure from the BMS-202-bound complex (PDB ID: 5J89) and chain A of the apo mouse PD-L1 structure (PDB ID: 6SRU) were aligned, and their IgV-domain backbones were structurally superposed. Pocket-proximal residues were identified with reference to the crystallographic human BMS-202-binding site and the published human-mouse PD-L1 comparison.

Because 6SRU contains a monomeric mouse PD-L1 IgV domain and no experimentally determined small-molecule-bound mouse PD-L1 dimer structure was available, a comparative mouse PD-L1 dimer model was constructed. Two copies of chain A from 6SRU were independently superposed onto the two ligand-associated PD-L1 protomers of the human 5J89 dimer. The human protein chains were then removed, yielding a 5J89-templated mouse PD-L1 dimer arrangement. The crystallographic ligand from 5J89, BMS-202 (PDB ligand ID: 6GX), was retained only as a spatial reference and was excluded from the docking receptor.

BMS-202, PD-L1-Ser, and PD-L1-Ser-OEt were prepared and docked to the modeled mouse PD-L1 dimer using the same AutoDock Vina receptor preparation, search region, and docking settings described for the human PD-L1 analysis. The top-ranked pose for each ligand was selected according to the Vina score and examined by three-dimensional visualization and two-dimensional interaction mapping. Because the mouse dimer geometry was imposed from the human 5J89 structure rather than experimentally determined, these calculations were used only for qualitative cross-species comparison and not for quantitative affinity estimation.

2. Tables

Supplementary Table S1. Reproducibility of AutoDock Vina docking scores across three independent random seeds.

Compound	Seed 101	Seed 202	Seed 303	Mean ± SD
BMS-202	-12.790	-12.920	-12.830	-12.847 ± 0.067

BMS-1001	-12.700	-12.650	-12.650	-12.667 ± 0.029
BMS-1166	-13.330	-13.350	-13.260	-13.313 ± 0.047
PD-L1-Ser	-12.204	-12.554	-12.297	-12.352 ± 0.181
PD-L1-Ser-OEt	-12.457	-12.509	-12.472	-12.479 ± 0.027

Note: Docking scores are reported in kcal/mol. Each compound was subjected to three independent AutoDock Vina runs using random seeds 101, 202, and 303 while all other docking parameters were kept unchanged. Values represent the best-ranked pose from each run. Docking scores were used for relative structure-based prioritization and were not quantitative binding affinities.

3. Figures

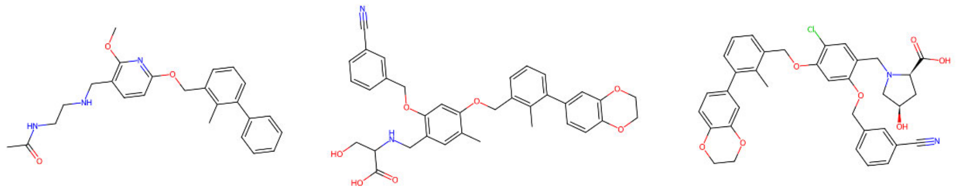

BMS-202
Reported HTRF IC50: 18 nM

BMS-1001
Reported HTRF IC50: 2.25 nM

BMS-1166
Reported HTRF IC50: 1.4 nM

Supplementary Figure S1. Chemical structures and reported HTRF inhibitory activities of representative BMS-series human PD-L1 inhibitors. The structures were redrawn from published chemical representations. Reported IC50 values are assay-specific and should not be interpreted as directly interchangeable across laboratories: BMS-202, 18 nM; BMS-1001, 2.25 nM; and BMS-1166, 1.4 nM [12-14].

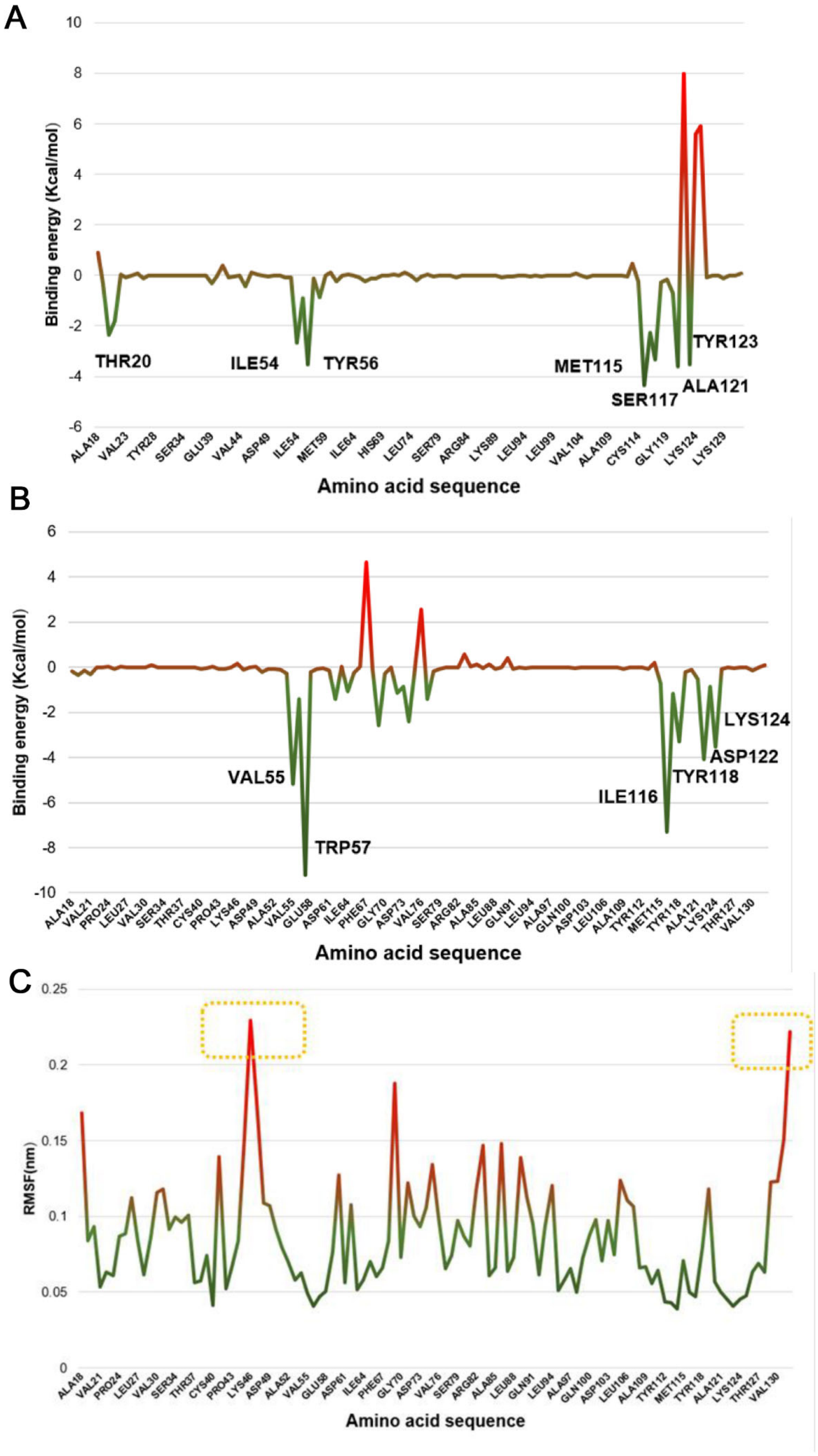
A
Binding energy (Kcal/mol)
THR20
ILE54
TYR56
MET115
SER117
TYR123
ALA121
Amino acid sequence
B
Binding energy (Kcal/mol)
VAL55
TRP57
ILE116
TYR118
ASP122
LYS124
Amino acid sequence
C
RMSF(nm)
Amino acid sequence

D

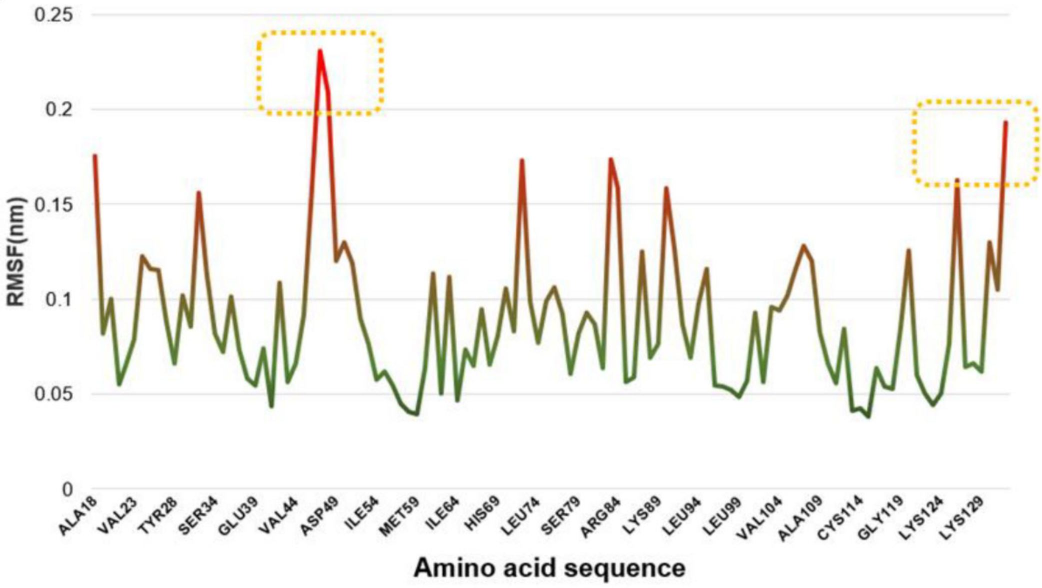

Supplementary Figure S2. MD analysis of the PD-L1 dimer bound to BMS202. (A-B) MM/PBSA binding-energy decomposition for BMS202 with the PD-L1 dimer: chain A (A) and chain B (B). (C-D) RMSF values of amino acid residues in chain A (C) and chain B (D).

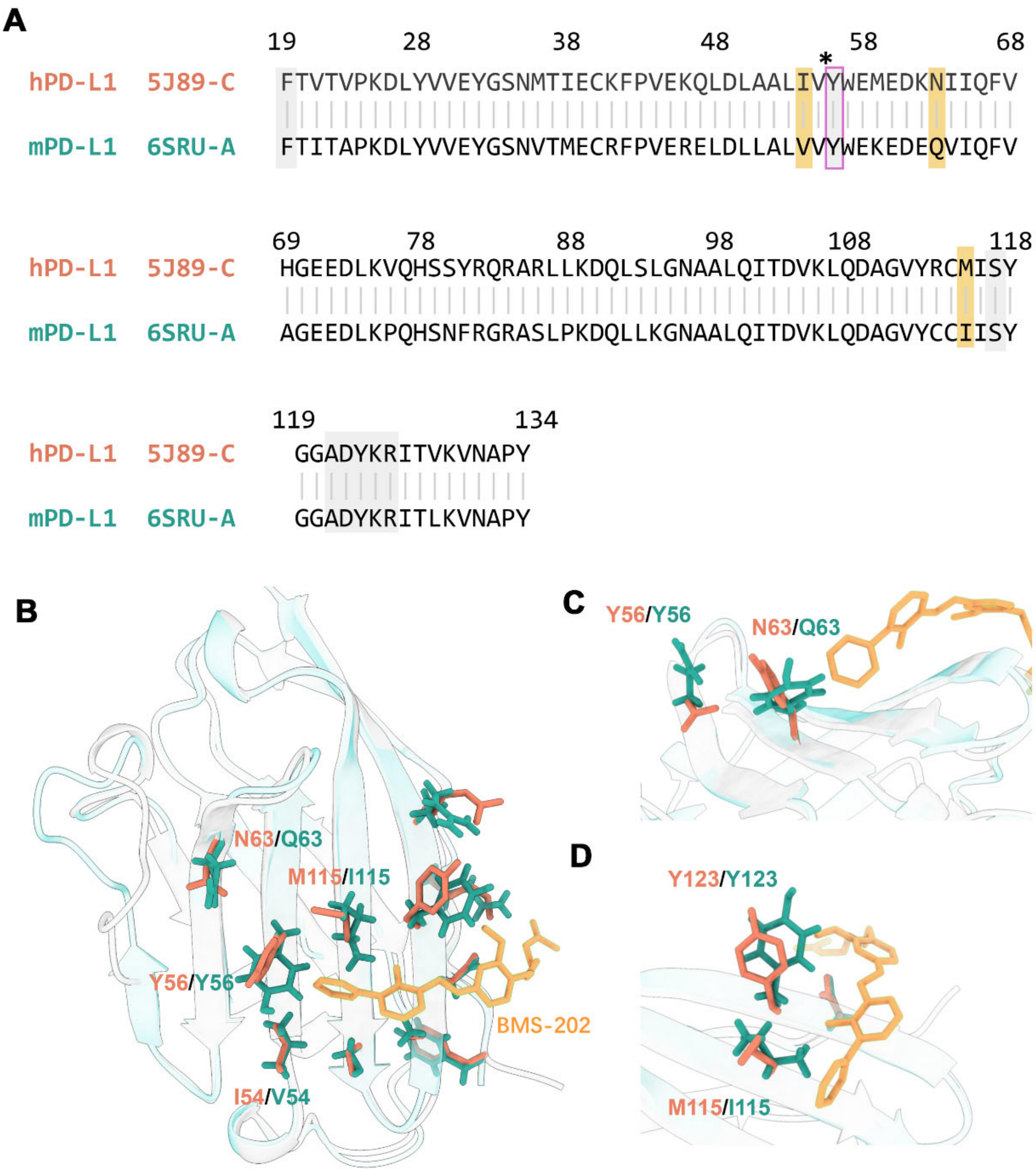

Supplementary Figure S3. Structural comparison of the proposed small-molecule binding region between human and mouse PD-L1. (A) Global structural superposition of the human PD-L1 IgV domain from the BMS-202-bound hPD-L1 complex (PDB ID: 5J89, chain C; gray) and the apo mouse PD-L1 IgV domain (PDB ID: 6SRU, chain A; cyan). The overall IgV folds are closely similar. (B) Comparison of the proposed small-molecule binding region. The human PD-L1 monomer from 5J89 (gray cartoon) and mouse PD-L1 monomer from 6SRU (cyan cartoon) were structurally superposed, and the co-crystallized ligand BMS-202 (6GX; orange sticks) from 5J89 is shown to indicate the canonical human small-molecule binding site. Pocket-proximal residues are displayed as sticks. Human residues are shown in coral and mouse residues in teal. (C) Zoomed-in view of the Tyr56/Asn63-Gln63 region. Tyr56 is conserved at the

sequence level in both species, but its side-chain orientation differs between the human and mouse structures. In addition, Gln63 in mouse PD-L1 has a longer side chain than Asn63 in human PD-L1, which may influence local conformational adaptability around Tyr56. (D) Zoomed-in view of the Met115/Ile115 region. The human Met115-to-mouse Ile115 substitution introduces a β-branched side chain into the hydrophobic channel and may increase local steric constraint. Conserved residues Asp122 and Tyr123 are shown as reference residues adjacent to the proposed binding site.

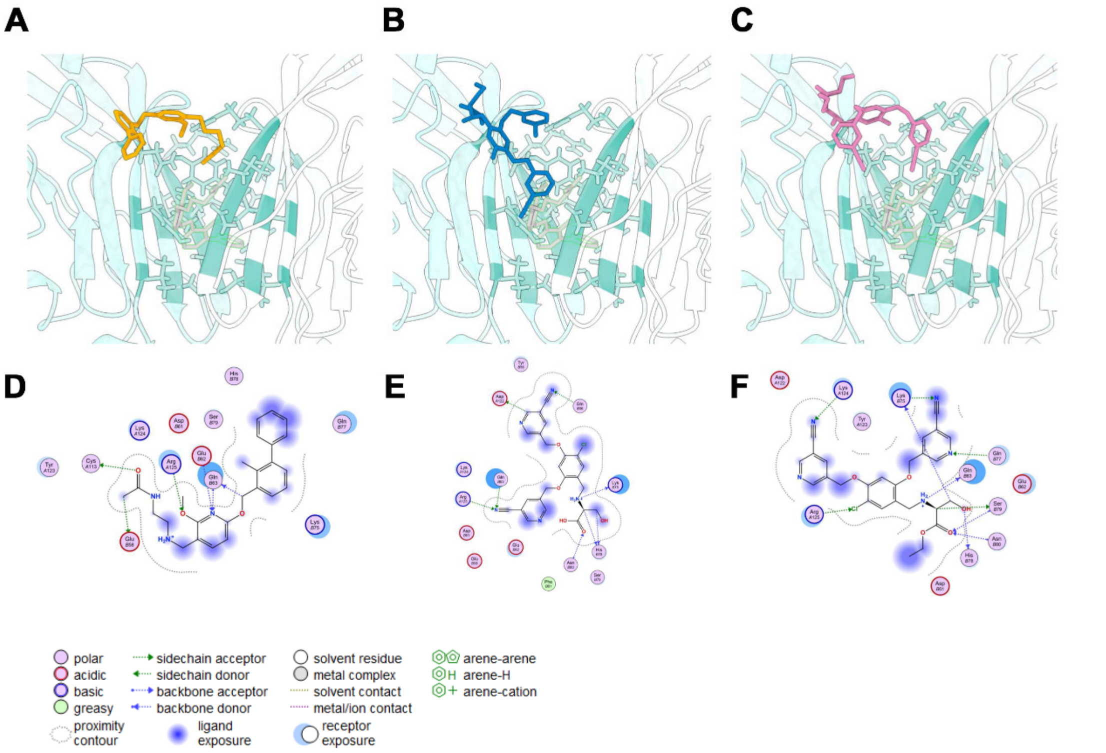

Supplementary Figure S4. Docking resultsof BMS-202, PD-L1-Ser, and PD-L1-Ser-OEt to a 5J89-templated mouse PD-L1 dimer model. A comparative mouse PD-L1 dimer model was generated by independently superposing two copies of the apo mouse PD-L1 IgV structure from 6SRU onto the two ligand-associated human PD-L1 protomers in 5J89. The modeled mouse PD-L1 dimer is shown as pale-cyan cartoons, and selected pocket-proximal residues are shown as teal sticks. The crystallographic position of BMS-202 in the human 5J89 structure is shown as semitransparent gray sticks in all panels to indicate the canonical human PD-L1 dimer-interface pocket. (A) Top-ranked AutoDock Vina pose of BMS-202 in the modeled mouse PD-L1 dimer, shown in orange. (B) Top-ranked pose of PD-L1-Ser, shown in blue. (C) Top-ranked pose of PD-L1-Ser-OEt, shown in magenta. (D-F) The interactions between mouse PD-L1 dimer and BMS-202 (D), PD-L1-Ser (E), or PD-L1-Ser-OEt (F), respectively. The

corresponding top-ranked Vina docking scores were -6.408, -6.404, and -5.895 kcal/mol for BMS-202, PD-L1-Ser, and PD-L1-Ser-OEt, respectively. The altered spatial locations of the modeled mouse poses relative to the crystallographic human BMS-202 position suggest potential species-dependent constraints within the proposed pocket.

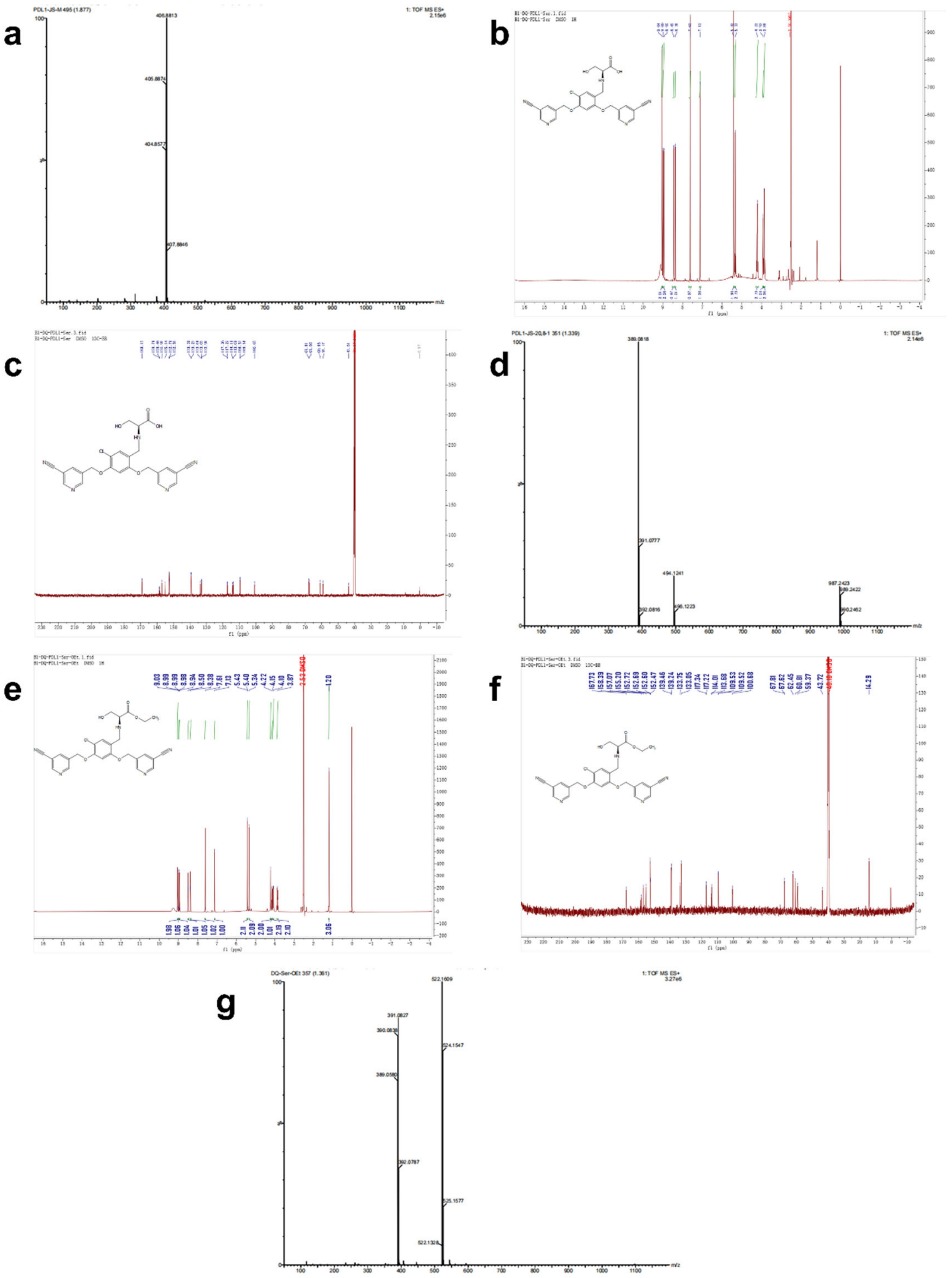

Supplementary Figure S5. Structural characterization of PD-L1-Ser and PD-L1-Ser-OEt. (**a**) MS spectrum of intermediate **3**. (**b**) ^{1}H NMR spectrum of PD-L1-Ser. (**c**) ^{13}C NMR spectrum of PD-L1-Ser. (**d**) MS spectrum of PD-L1-Ser. (**e**) ^{1}H NMR spectrum of PD-L1-Ser-OEt. (**f**) ^{13}C NMR spectrum of PD-L1-Ser-OEt. (**g**) MS spectrum of PD-L1-Ser-OEt.
